# Cumulative lipid–inflammatory indices and the risk of cardiometabolic multimorbidity: a prospective cohort study

**DOI:** 10.3389/fendo.2026.1909025

**Published:** 2026-08-10

**Authors:** Lijun Meng, Haibo Gao, Jing Yang, Shouling Wu, Shuohua Chen, Yuntao Wu, Qi Zhang

**Affiliations:** 1 Graduate School, Hebei Medical University, Shijiazhuang, Hebei, China; 2 Department of Cardiology, Affiliated Hospital of North China University of Science and Technology, Tangshan, Hebei, China; 3 Department of Cardiology, Tangshan Gongren Hospital, Tangshan, Hebei, China; 4 Department of Cardiology, Kailuan General Hospital, Tangshan, Hebei, China; 5 The Third Hospital of Hebei Medical University, Shijiazhuang, Hebei, China

**Keywords:** cardiometabolic multimorbidity, cumulative exposure, lipid-inflammatory index, prospective cohort study, risk prediction

## Abstract

**Background:**

Cardiometabolic multimorbidity (CMM) has become a major global public health challenge. Although dyslipidemia and chronic low-grade inflammation are known to contribute to cardiometabolic diseases, their long-term cumulative combined effects on the risk of CMM remain poorly understood. This study aimed to evaluate the associations of cumulative lipid-inflammatory indices with incident CMM in a large prospective cohort.

**Methods:**

A total of 25,376 participants from the Kailuan cohort were included and followed up until December 31, 2023. Nine cumulative lipid-inflammatory exposure indices were constructed via trapezoidal area-under-the-curve calculation using repeated measurements from 2006 to 2010. The primary outcome was incident CMM, defined as the concurrence of at least two cardiometabolic diseases including type 2 diabetes, ischemic heart disease and stroke. Multivariable Cox regression, restricted cubic spline analyses, competing-risk models, and subgroup analyses were applied.

**Results:**

During a median follow-up of 13.0 years, 481 incident CMM events were documented. All nine cumulative lipid-inflammatory indices were independently and positively associated with an elevated risk of CMM in a dose-response manner. Incorporating these indices into conventional risk model improved risk reclassification and discriminatory ability.

**Conclusions:**

Elevated cumulative lipid-inflammatory indices are associated with a higher risk of incident CMM. These composite markers may serve as auxiliary indicators for preliminary risk stratification, while causal relationships cannot be confirmed based on this observational cohort alone.

## Introduction

Cardiometabolic multimorbidity(CMM) is characterized by the concurrence of at least two cardiometabolic diseases (CMDs), including type 2 diabetes(T2D), ischemic heart disease(IHD) and stroke (1, 2). CMM represents a major global public health challenge that impairs physical and mental health, reduces quality of life, and drives high prevalence, disability, and mortality, while imposing substantial familial and societal healthcare burdens worldwide (3). Specifically, synergistic interactions between cumulative lipid abnormalities (e.g., high triglycerides and low high-density lipoprotein cholesterol) and persistent low-grade inflammation accelerate atherosclerosis and induce endothelial dysfunction, thereby promoting the development of diverse CMDs (4–6). From a molecular perspective, such lipid-inflammatory crosstalk disrupts endothelial homeostasis, promotes monocyte infiltration and foam cell formation, and triggers oxidative stress and cellular senescence, ultimately forming a vicious cycle that leads to progressive multisystem damage (5, 7).

Although the contributions of lipid disorders and inflammation to CMM are well recognized (8, 9), evaluating their long-term cumulative and synergistic effects remains challenging in large populations and clinical practice. Traditional risk assessment relies predominantly on single-time-point biomarker measurements (10), which cannot capture dynamic temporal changes in risk exposures and may substantially underestimate an individual’s true long-term risk. To overcome this limitation, novel indices that comprehensively reflect long-term cumulative lipid and inflammatory exposure (e.g., cumulative lipid-inflammatory indices) have been proposed and may confer stronger risk predictive power than single-time measurements (11). However, the validity of such cumulative indices-especially their ability to predict the incident of CMM-has not been systematically evaluated in large-scale prospective cohort studies.

The Kailuan Cohort in China is a large prospective investigation with long-term, standardized annual health examinations, providing a unique and well-established platform for calculating cumulative biomarker exposure (12, 13). Using this resource, the present study aimed to systematically investigate the longitudinal associations of a series of cumulative lipid-inflammatory indices with the risk of incident CMM. We anticipate our findings may provide auxiliary reference indicators for preliminary risk stratification of individuals with elevated CMM risk, as well as observational epidemiological evidence describing statistical correlations between cumulative lipid-inflammatory burden and CMM occurrence.

## Methods

### Study participants

In this study, we included participants who underwent consecutive health examinations in 2006, 2008, and 2010 (n=57,927). Exclusion criterias: (1)Individuals missed data for TG, TC, LDL-C, HDL-C, FBG, WT, HT or hs-CRP (n=15,948); (2)Individuals with a prior diagnosis of IHD、stroke or T2D (n=13,398); (3)Individuals with a prior diagnosis of cancer (n=509); (4)Individuals with acute infections (n=2,696). The current study’s total enrollment reached 25,376 participants. This study was approved by the Ethics Committee of Kailuan General Hospital (No. 200605). Written informed consent was obtained from all participants.

### Data collection and definitions

Epidemiological survey content and anthropometric measurements were collected according to our group’s previous literature (14). Biochemical measurement: Participants fasted for at least 8 hours prior to examination. Approximately 5 mL of fasting blood was drawn from the elbow vein the following morning. After centrifugation, the supernatant serum was collected for analysis. Biochemical indicators, including FBG, TG, HDL-C, LDL-C, TC, and hs-CRP, were measured using the Hitachi 7600 automatic biochemical analyzer (15). We measured FBG using the hexokinase assay and measured TG and HDL-C using the enzyme colorimetric assay. All tests were performed by professional laboratory technicians strictly following the manufacturer’s instructions. Smoking history definition: in the past year, the average number of cigarettes smoked at least 1 per day, lasting more than 1 year. Drinking history definition: the average daily drinking was more than 1 standard drinking volume (1 standard drinking volume is equivalent to 45ml liquor/360ml beer/120ml fruit wine), for more than 1 consecutive year, or has been abstained from alcohol for less than 1year. Physical exercise definition: ≥3 sessions per week, each lasting ≥30 min (16); BMI definition: weight (kg)/height² (m²) (17); Diagnoses of hypertension, diabetes, and hyperlipidemia followed Chinese guidelines (18–20). Missing covariate data were handled using multiple imputation.

### Exposures

The basic formula for lipid-inflammatory index was defined as follows (21–26):

lipid-inflammatory index = lipid index(mg/dL) *hs-CRP (mg/L)/10

In the following formulas, all lipid index and FBG are expressed in mg/dL, BMI in kg/m^2^ and hs-CRP in mg/L.

1. AIP_CRP= lg (TG/HDL-C)*hs-CRP/10;

2. CRI_I_CRP= (TC/HDL-C)*hs-CRP/10;

3. CRI_II_CRP=(LDL-C/HDL-C)*hs-CRP/10;

4. LCI_CRP= (TC × TG ×LDL-C/HDL-C)*hs-CRP/10;

5. NHHR_CRP=[(TC-HDL-C)/HDL-C]*hs-CRP/10

6. CHG_CRP = ln[(TC × FBG)/(2 × HDL-C)] × hs-CRP/10

7. LnRCII=ln(TC- HDL-C- LDL-C)*hs-CRP/10

8. TyHGB_CRP=(TG/HDL-C+0.7×FBG+0.1×BMI)*hs-CRP/10

9. CTI = 0.412 × ln(hs-CRP) + ln[(TG × FBG)/2]

10. To avoid reverse causality and ensure exposure assessment precedes outcome ascertainment, we set the baseline year as 2010 and quantified long-term cumulative lipid-inflammatory exposure from repeated measurements collected in 2006, 2008 and 2010 via the trapezoidal area-under-the-curve (AUC) approach. This trapezoidal formula yields total cumulative exposure (units: index-years):

cumulative lipid-inflammatory index = (index_2006_ +index_2008_)/2× time_2006-2008_+(index_2008_ + index_2010_)/2× time_2008-2010_

Time_2006–2008_ and time_2008–2010_ represent the time intervals (in years) between consecutive health examinations.

Note: all individuals with remnant cholesterol ≤ 0 were excluded when calculating LnRCII to avoid invalid logarithmic values.

### Follow-up period and outcomes

The follow-up period commenced with the 2010 physical examination. The primary endpoint of this study was the occurrence of CMM events, characterized by the coexistence of at least two CMDs, including stroke, IHD, and T2D (1, 2).The date of CMM incidence during the follow-up was defined as the date on which the second CMD diagnosis was confirmed. Follow-up time was calculated from the baseline physical examination date to the date of CMM incidence, death, or the censoring date (December 31, 2023), whichever came first. IHD、stroke and T2D diagnosis followed WHO criteria. IHD and stroke events information was obtained from the Kailuan Social Security Information System. Trained medical professionals collected data by reviewing records at Kailuan Group hospitals, with diagnoses confirmed by physicians based on inpatient medical records. Participants who reported a history of T2D or were treated for T2D and had a baseline FBG ≥ 7.0 mmol/L (126 mg/dL) or HbA1c ≥ 6.5% were defined as having T2D.

### Statistical analysis

The analysis of the data was conducted using SAS 9.4 software and R 4.2.2 statistical software. Missing covariate data were handled using multiple imputation. For quantitative variables, normally distributed continuous variables were expressed as mean ± standard deviation, with intergroup comparisons performed using ANOVA. The measurement data of non-normal distribution were expressed as M (*P*_25_, *P*_75_), and the non-parametric rank sum test was employed for the purpose of comparing the data across groups. Categorical variables were expressed as constituent ratios or rates (%), and χ² test was used for comparison among groups.

We described the distribution of four CMM phenotypic subtypes and visualized sequential disease transitions from healthy status to single cardiometabolic disease (CMD) and subsequent CMM using a flowchart. We evaluated collinearity among covariates using variance inflation factors (VIFs) computed for all terms included in each multivariable model. All VIF values were below 5, suggesting that significant multicollinearity among the covariates was unlikely. Multivariate Cox proportional hazards models were used to estimate CMM risk. Restricted cubic spline (RCS) regression model with four knots at the 5th, 35th, 65th and 95th percentiles was used to investigate the association between the cumulative lipid-inflammatory indices and the risk of CMM and its subtypes, and distinguish linear and nonlinear associations between cumulative lipid-inflammatory indices and incident CMM. Nonlinear indices were split at RCS cutoffs for two-piecewise Cox analysis.

The robustness of the correlation between the cumulative lipid-inflammatory indices and the incidence of CMM was also examined. Initially, cumulative lipid-inflammatory indices were incorporated into the model as a continuous variable. Secondly, given that the cohort comprises middle-aged and older adults, death during follow-up may preclude the occurrence or reporting of incident CMM. We therefore performed a competing-risk sensitivity analysis using the Fine-Gray subdistribution hazard model, treating all-cause death as a competing event and using the same adjustment variables as in the main model to repeat the analysis. Thirdly, to eliminate the possibility of reverse causality, the primary analyses were repeated, with participants who experienced CMM events during the initial 2 years of follow-up excluded from the analysis. Fourthly, we excluded participants who developed cancer during follow-up. Finally, considering the potential impact of lipid-lowering agents on the associations between indices and outcomes, we reassessed the associations after adjusting for these medications. Moreover, stratification analyses by age (≤60 vs>60 years), sex, BMI (≤24 vs>24 kg/m^2^), smoking (yes vs no), drinking (yes vs no), IGR (yes vs no) were performed.

To assess the discriminatory ability of these indices for predicting CMM, we constructed a conventional risk model including only the covariates in Model III and a novel model including the covariates in Model III and cumulative lipid-inflammatory indices. The net reclassification index (NRI) and integrated discrimination improvement index (IDI) were calculated by comparing the two models to assess whether adding cumulative lipid-inflammatory indices to conventional risk model could improve the predictive performance of CMM. Statistical significance was determined by a two-sided *P*<0.05.

## Results

### Baseline characteristics of study cohort

The characteristics of 25,376 participants stratified by CMM status are delineated in **Table 1**. Among them, 19,135(75.41%) were male, with a mean (SD) age of 52.07 ± 11.68 years. Compared with individuals who did not develop CMM, those who did were more likely to be older, male, smokers, have a higher prevalence of hypertension, hyperlipidemia, have higher usage rate of antihypertensive agents, lipid-lowering agents(all *P* < 0.05). Additionally, they exhibited higher baseline levels of BMI, WC, SBP, DBP, FBG, HR, TG, TC, LDL-C, UA, CR, hs-CRP, cumulative lipid-inflammatory indices, and lower HDL-C (all *P* < 0.05). We added **Supplementary Table 1** to display the count and proportion of missing data for each covariate. We constructed **Supplementary Table 2** to compare baseline demographic, anthropometric and metabolic indicators between participants excluded due to missing core lipid, glucose and inflammatory biomarkers (n=15,948) and the final analytical population (n=25,376).

**Table 1:** Baseline characteristics of participants with and without CMM.

Variable	Total	Non-CMM	CMM	*P* value
Age(years)	52.07 ± 11.68	52.00 ± 11.71	56.07 ± 9.35	<0.001
Male(%)	19,135(75.41)	18,725(75.22)	410(85.24)	<0.001
BMI(kg/m^2^)	24.60 ± 3.24	24.57 ± 3.23	26.24 ± 3.23	<0.001
WC(cm)	87.71 ± 10.21	87.61 ± 10.20	92.59 ± 9.52	<0.001
SBP(mmHg)	129.55 ± 18.88	129.34 ± 18.82	140.67 ± 18.83	<0.001
DBP(mmHg)	83.95 ± 10.79	83.85 ± 10.77	89.40 ± 10.58	<0.001
FBG(mg/dL)	94.62 ± 10.65	94.48 ± 10.57	102.09 ± 12.21	<0.001
HR(bpm)	72.84 ± 9.92	72.83 ± 9.92	73.15 ± 9.88	<0.001
TG(mg/dL)	111.57(77.03-168.24)	111.57(77.03-167.35)	140.79(95.63-211.62)	<0.001
TC(mg/dL)	195.56 ± 46.77	195.34 ± 46.88	206.65 ± 39.32	<0.001
HDL-C(mg/dL)	59.87 ± 16.71	59.95 ± 16.73	55.41 ± 15.13	<0.001
LDL-C(mg/dL)	102.00 ± 30.32	101.84 ± 30.23	110.51 ± 33.76	<0.001
UA(μmol/L)	300.25 ± 87.68	300.04 ± 87.65	311.17 ± 88.89	0.023
CR(μmol/L)	79.30 ± 21.34	79.20 ± 21.35	84.65 ± 20.14	<0.001
hs-CRP(mg/L)	1.19(0.60-2.42)	1.18(0.60-2.40)	1.64(0.90-3.35)	<0.001
cumAIP_CRP	0.53 ± 0.72	0.53 ± 0.71	0.90 ± 0.90	<0.001
cumCRI_I_CRP	2.52 ± 2.27	2.50 ± 2.26	3.48 ± 2.55	<0.001
cumCRI_II_CRP	1.25 ± 1.17	1.24 ± 1.16	1.77 ± 1.31	<0.001
cumLCI_CRP	39,068.06 ± 68,161.60	38,468.70 ± 67,427.44	70,089.23 ± 93,996.56	<0.001
cumNHHR_CRP	1.82 ± 1.79	1.81 ± 1.78	2.58 ± 2.04	<0.001
cumCHG_CRP	9.11 ± 7.13	9.06 ± 7.11	11.80 ± 7.67	<0.001
cumLnRCII	4.41 ± 4.61	4.37 ± 4.62	6.40 ± 4.00	<0.001
cumTyHGB_CRP	49.11 ± 39.05	48.74 ± 38.83	68.09 ± 45.03	<0.001
cumCTI	34.47 ± 4.45	34.43 ± 4.44	36.51 ± 4.38	<0.001
Exercise(%)	17,225(67.88)	16,894(67.86)	331(68.81)	0.906
Hypertension(%)	13,577(53.50)	13,168(52.89)	409(85.03)	<0.001
Hyperlipidemia(%)	16,883(66.53)	16,475(66.18)	408(84.82)	<0.001
Antihypertensive agents(%)	5,526(21.78)	5,273(21.18)	253(52.60)	<0.001
Lipid-lowering drugs(%)	1,635(6.44)	1,447(5.81)	188(39.92)	<0.001
Smoke(%)	8,777(34.59)	8,585(34.48)	192(39.92)	0.046
Drink(%)	9,353(36.86)	9,168(36.83)	185(38.46)	0.763

Data are presented as mean (SD) or median (interquartile range) for continuous variables and n (%) for categorical variables.

BMI, body mass index; WC, waist circumference; SBP, systolic blood pressure; DBP, diastolic blood pressure; FBG, fasting blood glucose; HR, heart rate; TG, triglyceride; TC, total cholesterol; HDL-C, high density lipoprotein cholesterol; LDL-C, low density lipoprotein cholesterol; UA, uric acid; CR, creatinine; hs-CRP, high-sensitivity C-reactive protein.

### Association between cumulative lipid-inflammatory indices and incident CMM

**Figure 1:**
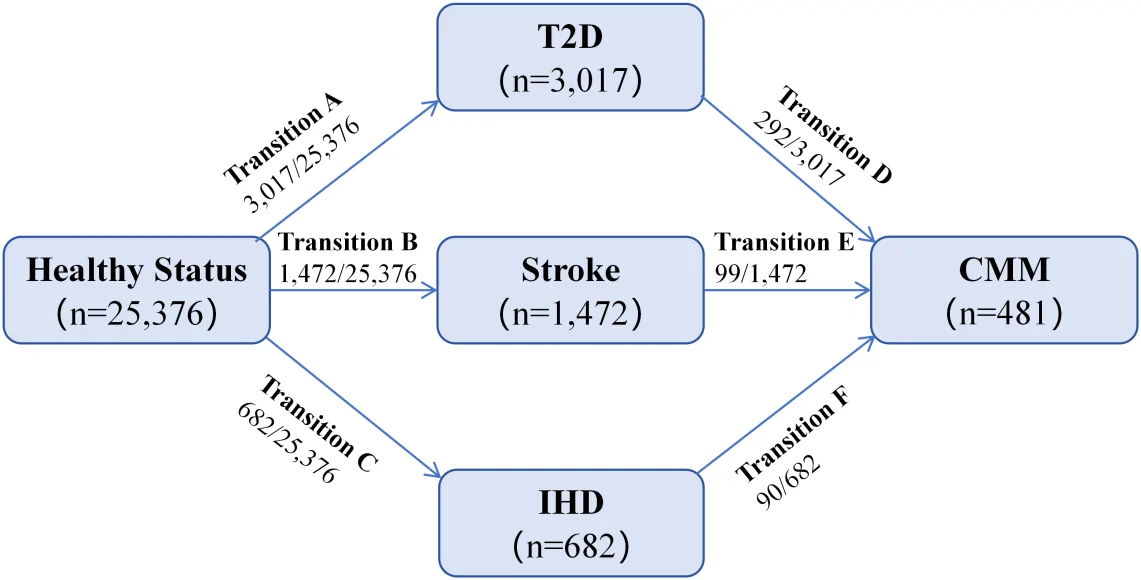
Multi-state transition patterns from baseline to CMM.

With a median follow-up duration of 13.00 (12.64, 13.31) years, a total of 481 incident cases of CMM (1.90% of the study cohort) were documented. The low incidence of CMM (1.90%) was consistent with the low event rate in healthy community-based cohorts with long follow-up. Among the 481 participants who developed incident CMM over follow-up, we further characterized the phenotypic composition of cardiometabolic multimorbidity combinations, with detailed case counts and proportional distributions of each subtype presented in **Table 2**. The most prevalent CMM phenotype was concurrent T2D and stroke, followed by T2D combined with IHD, IHD combined with stroke, and the co-occurrence of all three cardiometabolic diseases (T2D+IHD+stroke). **Table 2** revealed heterogeneity in the composition of CMM phenotypes within this community-based cohort. In addition, we visualized sequential disease transitions from healthy status to single CMD and subsequent CMM using a flowchart (**Figure 1**). This transition analysis demonstrated that a substantial proportion of incident CMM cases arose from pre-existing single CMD, highlighting the sequential natural history of cardiometabolic disorder accumulation in the study population.

**Table 2:** Distribution of four CMM phenotypic subtypes.

CMM subtype	Number of cases	Proportion among all CMM (%)
T2D + IHD	144	29.94
T2D + Stroke	268	55.72
IHD + Stroke	57	11.85
Triple comorbidity (T2D+IHD+Stroke)	12	2.49
Total CMM	481	100.00

In the multivariate Cox proportional hazards regression model (Model III), all nine cumulative lipid-inflammatory indices were significantly associated with an elevated risk of CMM in the highest tertile (**Table 3**). A significant positive trend was observed for all indices (all *P* for trend< 0.001), indicating a monotonically increasing risk of CMM with ascending tertile levels of the cumulative indices. The strongest associations were observed for cumLCI_CRP (HR = 2.27, 95% CI: 1.67-3.08), followed by cumAIP_CRP (HR = 1.89, 95%CI: 1.43-2.50) and cumTyHGB_CRP (HR = 1.85, 95%CI: 1.42-2.42). In contrast, the associations of these cumulative indices with individual cardiometabolic diseases (stroke, IHD and T2D) were substantially weaker. For transitions from the baseline “healthy” state to single CMDs, most cumulative lipid-inflammatory indices were significantly associated with these transitions, except for the transition from baseline to stroke. Among these associations, the strongest was observed with T2D, with HRs (95% CIs) of 2.03(1.82-2.26) for cumLCI_CRP and 1.79(1.62-1.97) for cumTyHGB_CRP. The second strongest association was with IHD, with HRs (95% CIs) of 1.50(1.23-1.83) for cumNHHR_CRP and 1.48(1.19-1.83) for cumLCI_CRP. For some indices (e.g., cumCTI and cumAIP_CRP), the HRs approached 1.00, with 95%CIs spanning the null value, indicating no statistically significant effects on individual CMD risk. Only two indices were significantly correlated with incident stroke in the multivariable adjustment model: cumCTI (HR = 1.18, 95%CI: 1.02-1.35) and cumAIP_CRP (HR = 1.16, 95%CI: 1.02-1.33) (**Supplementary Table 3**).

**Table 3:** HR for risk of CMM according to tertiles of the nine cumulative lipid-inflammatory indices.

Variables	Event(%)	HR(95%CI)		
		Model I	Model II	Model III
cumAIP_CRP				
Per 1 SD		1.34(1.28-1.40)	1.32(1.26-1.38)	1.18(1.10-1.26)
T1	75(0.89)	ref	ref	ref
T2	140(1.66)	1.88(1.42-2.49)	1.80(1.36-2.38)	1.31(0.98-1.74)
T3	266(3.14)	3.65(2.83-4.72)	3.37(2.61, 4.36)	1.89(1.43-2.50)
*P* for trend		<0.001	<0.001	<0.001
cumCRI_I_CRP				
Per 1 SD		1.26(1.21-1.32)	1.24(1.18-1.30)	1.14(1.06-1.22)
T1	80(0.95)	ref	ref	ref
T2	144(1.70)	1.83(1.40-2.41)	1.67(1.27-2.20)	1.27(0.96-1.67)
T3	257(3.04)	3.39(2.64-4.36)	2.94(2.28-3.78)	1.78(1.36-2.31)
*P* for trend		<0.001	<0.001	<0.001
cumCRI_II_CRP				
Per 1 SD		1.23(1.19-1.27)	1.22(1.17-1.26)	1.13(1.06-1.20)
T1	77(0.91)	ref	ref	ref
T2	149(1.76)	1.97(1.50-2.60)	1.80(1.37-2.37)	1.38(1.04-1.82)
T3	255(3.01)	3.51(2.72-4.53)	3.01(2.32-3.89)	1.79(1.37-2.35)
*P* for trend		<0.001	<0.001	<0.001
cumLCI_CRP				
Per 1 SD		1.08(1.06-1.10)	1.08(1.06-1.10)	1.07(1.04-1.10)
T1	61(0.72)	ref	ref	ref
T2	144(1.70)	2.40(1.78-3.24)	2.16(1.60-2.92)	1.55(1.14-2.11)
T3	276(3.26)	4.73(3.59-6.24)	4.19(3.18-5.54)	2.27(1.67-3.08)
*P* for trend		<0.001	<0.001	<0.001
cumNHHR_CRP				
Per 1 SD		1.21(1.17-1.25)	1.20(1.15-1.25)	1.12(1.06-1.20)
T1	77(0.91)	ref	ref	ref
T2	144(1.70)	1.90(1.44-2.51)	1.73(1.31-2.28)	1.28(0.97-1.69)
T3	260(3.07)	3.56(2.76-4.60)	3.08(2.38-3.98)	1.83(1.40-2.39)
*P* for trend		<0.001	<0.001	<0.001
cumCHG_CRP				
Per 1 SD		1.38(1.29-1.48)	1.31(1.21-1.41)	1.14(1.05-1.24)
T1	87(1.03)	ref	ref	ref
T2	158(1.87)	1.86(1.43-2.41)	1.70(1.30-2.20)	1.31(1.01-1.71)
T3	236(2.79)	2.86(2.23-3.65)	2.48(1.93-3.18)	1.56(1.21-2.02)
*P* for trend		<0.001	<0.001	<0.001
cumLnRCII				
Per 1 SD		1.67(1.51-1.84)	1.57(1.42-1.74)	1.25(1.12-1.39)
T1	83(0.98)	ref	ref	ref
T2	141(1.67)	1.71(1.31-2.25)	1.59(1.21-2.09)	1.20(0.91-1.59)
T3	257(3.04)	3.22(2.52-4.13)	2.82(2.20-3.62)	1.69(1.30-2.20)
*P* for trend		<0.001	<0.001	<0.001
cumTyHGB_CRP				
Per 1 SD		1.47(1.37-1.57)	1.40(1.30-1.50)	1.21(1.12-1.31)
T1	76(0.90)	ref	ref	ref
T2	151(1.79)	2.03(1.54-2.58)	1.85(1.40-2.44)	1.39(1.05-1.83)
T3	254(3.00)	3.53(2.73-4.56)	3.04(2.35-3.94)	1.85(1.42-2.42)
*P* for trend		<0.001	<0.001	<0.001
cumCTI				
Per 1 SD		1.62(1.49-1.75)	1.49(1.36-1.63)	1.23(1.11-1.36)
T1	77(0.91)	ref	ref	ref
T2	160(1.89)	2.19(1.67-2.87)	1.88(1.43-2.48)	1.38(1.04-1.82)
T3	244(2.88)	3.60(2.78-4.66)	2.78(2.13-3.64)	1.69(1.28-2.24)
*P* for trend		<0.001	<0.001	<0.001

Model I: non-adjusted.

Model II: adjusted for age, sex.

Model III: adjusted for Model II+BMI, SBP, DBP, WC, HR, CR, UA, hypertension, hyperlipidemia, exercise, drink, smoke, and medication use (antihypertensive agents, lipid-lowering drugs).

**Figure 2:**
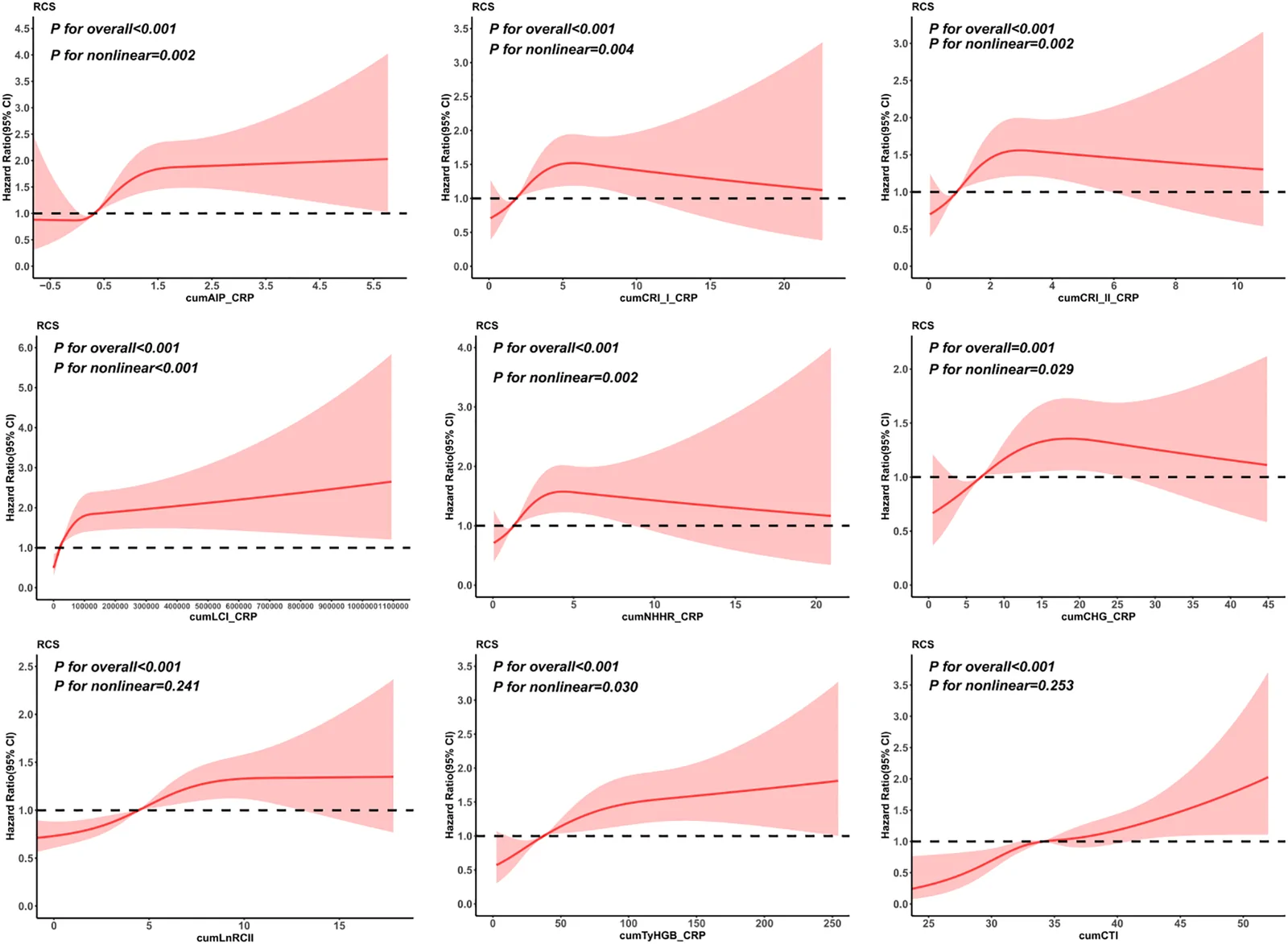
Dose-response relationships between nine cumulative lipid-inflammatory indices and CMM risk. Models were adjusted for age, sex, BMI, SBP, DBP, WC, HR, CR, UA, hypertension, hyperlipidemia, exercise, drink, smoke, and medication use (antihypertensive agents, lipid-lowering drugs).

Restricted cubic spline analyses further revealed distinct dose-response relationships between the nine cumulative lipid-inflammatory indices and specific cardiometabolic outcomes. For CMM (**Figure 2**), most of these indices exhibited significant positive nonlinear associations (*P* for overall< 0.05; *P* for nonlinear< 0.05), whereas cumCTI and cumLnRCII displayed significant positive linear associations (*P* for overall< 0.05; *P* for nonlinear> 0.05). For incident stroke, only cumLCI_CRP presented significant overall effects (*P* for overall<0.05) and significant nonlinearity (*P* for nonlinear <0.05), while the remaining eight indices showed non-significant overall associations. For IHD, significant overall associations were detected for all indices except cumCTI; among these, cumAIP_CRP, cumCRI_I_CRP, cumCRI_II_CRP, cumLCI_CRP and cumNHHR_CRP exhibited significant nonlinear relationships (*P* for nonlinear< 0.05), whereas cumCHG_CRP, cumLnRCII and cumTyHGB_CRP followed linear patterns (*P* for nonlinear> 0.05). Similar patterns were observed for T2D: all eight indices showed nonlinear associations with T2D, with the sole exception of cumLnRCII, which was linearly associated with T2D risk (**Supplementary Figures 1**-**S3**).

Restricted cubic spline analyses distinguished linear and nonlinear associations between cumulative lipid-inflammatory indices and incident CMM. Except for linear indices cumLnRCII and cumCTI, the other seven indices exhibited a consistent nonlinear association with incident CMM. Below the identified inflection breakpoint, each unit increment in the index was associated with higher CMM risk. In contrast, after exceeding the threshold value, further elevation of the lipid-inflammatory index was not linked to additional risk increment. Likelihood ratio tests verified the existence of threshold effects for all seven indicators (**Table 4**).

**Table 4:** Two-piecewise Cox regression for CMM stratified by RCS-derived breakpoints.

Variable	HR(95%CI)	*P* value
The inflection point of cumAIP_CRP	1.31	
< 1.31	1.83(1.46-2.29)	<0.001
> 1.31	1.00(0.84-1.20)	0.990
*P* for likelihood test	<0.001	
The inflection point of cumCRI_I_CRP	3.27	
< 3.27	1.27(1.14-1.42)	<0.001
> 3.27	1.00(0.95-1.05)	0.943
*P* for likelihood test	<0.001	
The inflection point of cumCRI_II_CRP	2.09	
< 2.09	1.48(1.26-1.75)	<0.001
> 2.09	0.98(0.88-1.09)	0.690
*P* for likelihood test	<0.001	
The inflection point of cumLCI_CRP	98991.14	
< 98991.14	1.54(1.30-1.82)	<0.001
> 98991.14	1.04(0.71-1.52)	0.864
*P* for likelihood test	<0.001	
The inflection point of cumNHHR_CRP	2.28	
< 2.28	1.41(1.21-1.65)	<0.001
> 2.28	1.01(0.96-1.07)	0.709
*P* for likelihood test	<0.001	
The inflection point of cumCHG_CRP	23.50	
< 23.50	1.03(1.02-1.05)	<0.001
> 23.50	0.95(0.90-1.00)	0.057
*P* for likelihood test	0.004	
The inflection point of cumTyHGB_CRP	47.30	
< 47.30	1.02(1.01-1.03)	<0.001
> 47.30	1.00(1.00-1.01)	0.047
*P* for likelihood test	0.005	

Adjusted for age, sex, BMI, SBP, DBP, WC, HR, CR, UA, hypertension, hyperlipidemia, exercise, drink, smoke, and medication use (antihypertensive agents, lipid-lowering drugs).

### Predictive performance of nine cumulative lipid-inflammatory indices for CMM

We further evaluated the incremental predictive value of adding each cumulative lipid-inflammatory index to the conventional clinical risk model for incident CMM. The C-index only slightly rose from 0.7977 of the baseline clinical model to a narrow range of 0.7995-0.8027 across extended models, which indicated a modest overall improvement in global discrimination. Despite this small absolute increment in C-index, all extended models achieved statistically significant category-free NRI (all *P* < 0.05), reflecting meaningful individual risk reclassification. The largest NRI increments were observed for cumNHHR_CRP (0.2144), cumTyHGB_CRP (0.2104), and cumLnRCII (0.2097). Statistically significant IDI increments were only detected for models incorporating cumAIP_CRP, cumLnRCII and cumLCI_CRP; the remaining indices improved risk stratification but showed non-significant IDI changes (**Table 5**).

**Table 5:** Incremental predictive value of nine cumulative lipid-inflammatory indices for CMM.

Risk models	C-index (95% CI)	Category-free NRI (95% CI)	*P* value	IDI (95% CI)	*P* value
Conventional risk model	0.7977(0.7786,0.8167)				
Conventional risk model +cumAIP_CRP	0.8009(0.7818,0.8200)	0.1718(0.0822,0.2615)	0.0002	0.0011(0.0002,0.0021)	0.0173
Conventional risk model +cumCRI_I_CRP	0.8001(0.7811,0.8191)	0.1877(0.0981,0.2772)	<0.0001	0.0004(-0.0002,0.0011)	0.1806
Conventional risk model +cumCRI_II_CRP	0.8008(0.7819,0.8198)	0.1861(0.0965,0.2757)	<0.0001	0.0004(-0.0003,0.0010)	0.2530
Conventional risk model +cumLCI_CRP	0.7995(0.7805,0.8185)	0.1409(0.0521,0.2298)	0.0022	0.0005(0.0001,0.0009)	0.0128
Conventional risk model +cumNHHR_CRP	0.7999(0.7809,0.8190)	0.2144(0.1247,0.3042)	<0.0001	0.0005(-0.0002,0.0011)	0.1508
Conventional risk model +cumCHG_CRP	0.8002(0.7812,0.8192)	0.1870(0.0972,0.2767)	<0.0001	0.0004(-0.0003,0.0010)	0.2937
Conventional risk model +cumLnRCII	0.8006(0.7815,0.8197)	0.2097(0.1201,0.2993)	<0.0001	0.0009(0.0002,0.0016)	<0.0001
Conventional risk model +cumTyHGB_CRP	0.8027(0.7838,0.8217)	0.2104(0.1206,0.3002)	<0.0001	0.0009(-0.0001,0.0020)	0.0831
Conventional risk model +cumCTI	0.7998(0.7807,0.8188)	0.1271(0.0369,0.2173)	0.0004	0.0007(-0.0001,0.0014)	0.0833

The conventional risk model included age, sex, BMI, SBP, DBP, WC, HR, CR, UA, hypertension, hyperlipidemia, exercise, drink, smoke, and medication use (antihypertensive agents, lipid-lowering drugs).

### Subgroup analysis

Subgroup analyses were conducted to assess the consistency of associations across strata of age (≤60 vs >60 years), sex, BMI (≤24 vs >24 kg/m²), smoking, drinking, and IGR. For all cumulative lipid-inflammatory indices, the positive associations with incident CMM remained generally consistent across most subgroups, especially among participants older than 60 years, female participants, those with BMI >24 kg/m², non-smokers, non-drinkers, and individuals with IGR (all *P* < 0.05). Significant effect modifications by IGR status were consistently observed across all indices (*P* for interaction < 0.001). Significant interactions were also detected for sex and BMI in several indices, whereas age, smoking and drinking showed no or weak interaction (mostly *P* for interaction> 0.05). The associations were generally stronger in female participants and those with higher BMI, supporting the robustness of these indices for CMM risk stratification in relevant subgroups (**Supplementary Figure 4**).

### Sensitivity analysis

Sensitivity analyses were performed to verify the robustness of the primary findings. First, competing-risk analyses were conducted using the Fine-Gray subdistribution hazard model, with all-cause mortality treated as a competing event. Second, participants who developed CMM during the first 2 years of follow-up were excluded to minimize reverse causality. Third, individuals diagnosed with cancer during follow-up were excluded. Finally, we further adjusted for baseline use of lipid-lowering medications to account for their potential confounding effects.

Overall, the magnitude and direction of the associations between cumulative lipid-inflammatory indices and incident CMM remained materially unchanged across all sensitivity analyses. These consistent results confirmed the reliability and stability of the main findings, indicating that elevated cumulative lipid-inflammatory indices were robustly associated with a higher risk of incident CMM (**Supplementary Tables 5**, **S6**).

## Discussion

This large-scale prospective cohort study comprehensively investigated the associations of nine cumulative lipid-inflammatory indices with the risk of incident CMM. Restricted cubic spline analyses demonstrated that all nine cumulative indices were independently and positively associated with an increased risk of CMM in a dose-dependent manner. Furthermore, adding these indices to conventional risk models improved risk reclassification and discrimination, as reflected by increases in NRI and IDI, supporting their incremental value in clinical CMM risk stratification. This study provides novel epidemiological evidence supporting cumulative pathogenic contribution of lipid dysmetabolism and chronic inflammation to CMM, and validates these cumulative indices as more reliable long-term predictors than traditional single-time-point measurements.

As summarized by a series of multinational prospective cohort studies, the vast majority of existing relevant research only relies on one-time laboratory testing to establish cross-sectional or single-wave longitudinal associations between IR-inflammatory indices and single cardiovascular endpoints, ignoring the persistent, progressive damage caused by years of abnormal lipid and inflammatory metabolism (27–31). Static single biomarkers can only reflect metabolic status at a certain snapshot, failing to capture the dynamic, accumulating pathological burden that drives the stepwise progression of multiple concurrent cardiometabolic disorders. Supported by causal evidence regarding triglyceride-related metabolic dysfunction, our cumulative indicators effectively overcome the static limitations of single biomarkers by integrating long-term persistent metabolic stress, thereby achieving superior risk discrimination for composite cardiometabolic outcomes (32). Given that statin therapy has been proven effective for cardiovascular protection in individuals with elevated hs-CRP levels (33), our results imply that dual interventions targeting lipid disorders and inflammation may represent a supplementary strategy for attenuating atherosclerosis and reducing CMM risk. Nevertheless, the overall incremental discrimination of these cumulative indices remains modest, indicating that their clinical value for large-scale precise CMM prevention should be further validated.

This study observed that all cumulative lipid-inflammation indices were associated with the risk of CMM, and the strength of this association was far greater than their association with individual diseases (such as IHD or stroke). This difference likely stems from the fundamental etiological differences between CMM and individual diseases. CMM is understood as a state in which multiple diseases coexist within the same individual, potentially driven by shared pathophysiological pathways—a “common ground” (34). Long-term accumulation of lipid abnormalities and chronic inflammation are the core components of this “common ground” (21, 35–37).The cumulative indices used in this study, by integrating data from multiple years, quantify this long-term, synergistic burden of metabolic and inflammatory dysregulation. Cumulative indices better reflect this persistent biological damage process than single-point measurements (38–40). Consequently, they are naturally better suited to identifying individuals exposed to high-risk “common ground” who are therefore more likely to develop CMM, rather than merely predicting single-endpoint events driven by other specific or acute factors (such as plaque rupture or arrhythmia). They capture the upstream, long-term, and comprehensive risks leading to multisystem damage, rather than the downstream, immediate, and specific manifestations of a single disease. The CMM subtype distribution and disease transition flowchart illustrate that long-term cumulative lipid-inflammatory burden preferentially accelerates the accumulation of multiple cardiometabolic disorders rather than triggering isolated single cardiometabolic lesions alone.

While both the lipid and hs-CRP are related to CMM, each has limitations as a standalone measure: lipid does not reflect inflammation, and hs-CRP alone does not capture metabolic dysregulation. The co-occurrence of lipid metabolic disturbances and persistent inflammation constitutes a key mechanism underlying cardiovascular disease development, as supported by current evidence (41). According to report, lipid abnormalities may further exacerbate disease risk by activating inflammatory pathways (42). Lipid metabolism dysregulation establishes a lipotoxic microenvironment characterized by FFAs, ceramides, and oxidized lipids. Within this microenvironment, coordinated innate or adaptive immune activation propagates metabolic inflammation, heightening circulating inflammatory mediators including hs-CRP. Furthermore, hs-CRP may disrupt leptin signaling, leading to leptin resistance, exacerbated adiposity, and further inflammation-creating a vicious cycle that accelerates metabolic and cardiovascular dysfunction (43). Hs-CRP also facilitates atherosclerosis by forming complexes with ox-LDL and promotes insulin resistance by impairing β-cell function and insulin signaling (44, 45). These cumulative lipid-inflammatory indices are predominantly composite indicators that better reflect the overall vascular burden of lipid metabolic disorders and inflammation compared to single lipid markers and inflammatory markers. Huang et al. confirmed (21) that dynamic monitoring of cumulative lipid-inflammatory indices demonstrated significant associations with higher stroke risk, with long-term accumulated elevations in these indices reflecting sustained vascular wall injury and increased systemic inflammatory burden, embodying the temporal synergistic interaction between lipid metabolic disorders and inflammatory responses, which explains their superior predictive value for CMM risk observed in our study.

Inter-individual variability in the predictive power of cumulative indices is jointly shaped by fat distribution patterns and inherited metabolic predisposition (46, 47). Subcutaneous fat exhibits lipid-buffering protective effects, whereas visceral adiposity aggravates systemic inflammatory cascades, partially explaining the stronger predictive associations observed in centrally obese individuals (46). Large-scale UK Biobank data further confirmed an additive interaction between polygenic cardiometabolic susceptibility and insulin resistance burden, such that individuals with high genetic metabolic risk exhibit synergistically elevated CMM susceptibility under sustained metabolic dysregulation (47). This gene-adipose interaction supports stratified prevention strategies, suggesting that individuals with central obesity or high genetic predisposition require regular longitudinal monitoring of lipids, glucose, and inflammatory markers to track progressive metabolic deterioration. Consistent with adipose remodeling mechanisms, lifestyle modification and GLP-1 agonists effectively reduce visceral fat accumulation and suppress systemic inflammation, thereby slowing the elevation of cumulative lipid-inflammatory indices (48). Thiazolidinediones also improve whole-body insulin sensitivity by redistributing lipid storage from visceral to subcutaneous adipose tissue, independent of body weight change. Such targeted lifestyle and pharmacological interventions help interrupt the self-perpetuating metabolic and inflammatory cycles that drive progressive cardiometabolic multimorbidity.

Among the nine cumulative lipid-inflammatory indices examined, cumLCI_CRP, cumAIP_CRP, and cumTyHGB_CRP showed the strongest statistical associations with incident CMM among all composite markers, with only modest auxiliary value for risk screening. First, cumLCI_CRP exhibited the most robust association among all nine cumulative lipid-inflammatory indices. This index integrates comprehensive atherogenic lipid profiles together with hs-CRP to fully reflect long-term synergistic lipid and inflammatory burden. It reliably captures synergistic interactions between atherogenic lipids and systemic inflammation that drive atherosclerotic progression (45). CumAIP_CRP delivered effective risk stratification ability among all nonlinear markers. Below its inflection point of 1.31, each unit elevation was linked to elevated CMM risk, which enables clear differentiation of individuals with mild-to-moderate metabolic-inflammatory burden, reliably captured lipid particle abnormality and low-grade inflammation, it is stable in calculation, easy to implement, and highly suitable for clinical application (49). CumTyHGB_CRP achieved the maximum improvement in overall model discrimination and prominent net reclassification improvement. It integrates lipid, glucose, BMI, and inflammatory parameters, and closely reflects the core pathological characteristics driving cardiometabolic multimorbidity (50).

These cumulative lipid-inflammatory indices deliver statistically significant yet modest incremental predictive performance for early risk screening and auxiliary risk-guided management of CMM (11, 51). By using long-term average exposure, these indices overcome the instability of single-time-point measurements and more accurately reflect persistent metabolic-inflammatory burden². From an intervention perspective, single lipid-lowering management fails to block the vicious cycle linking dyslipidaemia, chronic low-grade inflammation and insulin resistance. Dual intervention strategies combining lipid regulation and anti-inflammatory therapy are preferred for people with elevated cumulative lipid-inflammatory burden to hinder the progression toward incident CMM. This dual-target prevention strategy is conducive to realizing prevention of cardiometabolic multimorbidity and lowering the population-level disease burden, especially for overweight/obese patients and subjects with impaired glucose regulation. Therefore, incorporating these simple, low-cost, and easy-to-obtain biomarkers into routine physical examination can improve early identification of high-risk individuals and help achieve prevention of CMM at the population level (52). Conventional single lab tests delay early risk detection and exacerbate the global disease burden (53); our low-cost cumulative indices are suitable for community screening. Integrated cardiometabolic interventions can slow the growth of cumulative lipid burden, while high-index populations need intensive glycemic monitoring under stress (48, 54). Incorporating these cumulative composite indices into routine physical examination may provide supplementary correlative information to assist preliminary risk classification in asymptomatic participants.

This study has several limitations that should be acknowledged. First, this research relies on an observational prospective cohort design, multivariate Cox regression and multiple sensitivity analyses cannot completely eliminate residual confounding, so definitive causal relationships between cumulative lipid-inflammatory indices and CMM risk cannot be established merely based on the current dataset. All observed statistical associations do not equal causal pathogenic effects. Further verification through large-scale prospective studies and randomized controlled trials are warranted to confirm causality. Second, although we adjusted for a wide range of potential confounders, residual confounding cannot be entirely excluded, particularly from unmeasured factors such as detailed dietary intake, family history of cardiovascular disease, medication adherence, and underlying chronic inflammatory disorders or immunomodulatory drug use. Third, because distinct stroke subtypes may have divergent pathophysiological mechanisms and risk profiles, our lack of subtype classification limited the interpretability of stroke-related findings. Forth, the use of baseline medication status rather than time-varying information may introduce residual confounding, as lipid-lowering and antihypertensive may change during follow-up and influence both exposure and outcome. Fifth, the generalizability of our results is restricted by the characteristics of the Kailuan cohort, which comprises predominantly middle-aged and elderly male industrial workers from northern China. Our study sample mainly consists of northern Chinese middle-aged and elderly male industrial workers (75.41% male). Thus, results cannot be directly extrapolated to females, young adults, non-industrial residents, people from other regions or ethnic groups. Multi-ethnic and multi-center cohorts are required to improve external validity. Future studies involving multi-ethnic, multi-center populations are needed to enhance external validity. Sixth, future independent cohort studies with dedicated model validation designs are needed to evaluate the calibration degree, clinical net benefit and internal stability of these composite indices via DCA and bootstrap cross-validation. Finally, only hs-CRP was used as an inflammatory marker in this study; other key inflammatory cytokines such as IL-6 and TNF-α may also participate in the inflammatory cascade leading to CMM. Further studies with a more comprehensive inflammatory panel are warranted to clarify the complex inflammatory mechanisms underlying CMM.

## Conclusions

In summary, elevated cumulative lipid-inflammatory indices are positively associated with incident CMM within this cohort of northern Chinese middle-aged male industrial workers. These composite indicators can only provide supplementary correlative information for preliminary risk classification. The observational associations reported here cannot support causal inference or population-wide precision preventive interventions, and further multi-population and interventional studies are needed to validate their clinical utility.

## Data Availability

The original contributions presented in the study are included in the article/**Supplementary Material**. Further inquiries can be directed to the corresponding author.

## References

[1] Han Y Hu Y Yu C Sun D Pang Y Pei P . Duration-dependent impact of cardiometabolic diseases and multimorbidity on all-cause and cause-specific mortality: a prospective cohort study of 0.5 million participants. Cardiovasc Diabetol. (2023) 22. doi: 10.1186/s12933-023-01858-9 37308998 PMC10262418

[2] Shi S Zhou F Shen J . Trends in the prevalence of cardiometabolic diseases in US adults with newly diagnosed and undiagnosed diabetes, 1988–2020. Public Health. (2025) 239:94–102. doi: 10.1016/j.puhe.2024.12.041 39799659

[3] Su B Liu C Chen L Wu Y Li J Zheng X . Long-term exposure to PM2.5 and O3 with cardiometabolic multimorbidity: evidence among Chinese elderly population from 462 cities. Ecotoxicology Environ Saf. (2023) 255. doi: 10.1016/j.ecoenv.2023.114790 36948004

[4] Hotamisligil GS . Inflammation, metaflammation and immunometabolic disorders. Nature. (2017) 542:177–85. doi: 10.1038/nature21363 28179656

[5] Libby P Buring JE Badimon L Hansson GK Deanfield J Bittencourt MS . Atherosclerosis. Nat Rev Dis Primers. (2019) 5. doi: 10.1016/b978-0-7216-0284-4.50012-9 31420554

[6] Lavi S Bae JH Rihal CS Prasad A Barsness GW Lennon RJ . Segmental coronary endothelial dysfunction in patients with minimal atherosclerosis is associated with necrotic core plaques. Heart. (2009) 95:1525–30. doi: 10.1136/hrt.2009.166017 19497916 PMC4360895

[7] Gimbrone MA Jr Garcia-Cardena G . Endothelial cell dysfunction and the pathobiology of atherosclerosis. Circ Res. (2016) 118:620–36. doi: 10.1096/fasebj.22.1_supplement.471.11 PMC4762052 26892962

[8] Li Z Zhong B Xu W Sun L Hu X . Cumulative inflammatory index and the risk of cardiometabolic multimorbidity: a prospective cohort study among middle-aged and older adults in China. J Health Popul Nutr. (2026) 45. doi: 10.1186/s41043-026-01289-8 41866500 PMC13130790

[9] Chu NHS Wang H Yang M Cai W Zeng H Luo X . C-reactive protein-triglyceride-glucose index versus triglyceride-glucose index in predicting cardiovascular metabolic multimorbidity risk: a cohort study. PloS One. (2026) 21. doi: 10.1371/journal.pone.0340098 PMC12880693 41649995

[10] Pradhan AD Cook NR Buring JE Manson JE Ridker PM . C-reactive protein is independently associated with fasting insulin in nondiabetic women. Arterioscler Thromb Vasc Biol. (2003) 23:650–5. doi: 10.1161/01.atv.0000065636.15310.9c 12692007

[11] Zhang W Jing C Liu X Hou H . Cumulative average remnant cholesterol inflammatory index and risk of cardiometabolic multimorbidity: evidence from a prospective nationwide cohort study in China. Lipids Health Dis. (2026) 25:41. doi: 10.1186/s12944-025-02850-w 41501821 PMC12879387

[12] Wu S An S Li W Lichtenstein AH Gao J Kris-Etherton PM . Association of trajectory of cardiovascular health score and incident cardiovascular disease. JAMA Netw Open. (2019) 2:e194758. doi: 10.1001/jamanetworkopen.2019.4758 31150075 PMC6547110

[13] Wang C Yuan Y Zheng M Pan A Wang M Zhao M . Association of age of onset of hypertension with cardiovascular diseases and mortality. J Am Coll Cardiol. (2020) 75:2921–30. doi: 10.1007/978-981-15-0591-1_12 32527401

[14] Wu S Huang Z Yang X Zhou Y Wang A Chen L . Prevalence of ideal cardiovascular health and its relationship with the 4-year cardiovascular events in a northern Chinese industrial city. Circ Cardiovasc Qual Outcomes. (2012) 5:487–93. doi: 10.1161/circoutcomes.111.963694 22787064

[15] Liu T Wang S Song Q Liu A Du Y Wu S . Impact of cumulative exposure to fasting plasma glucose on cardiovascular and cerebrovascular events. Chin J Hypertens. (2022) 30:244–52. doi: 10.16439/j.issn.1673-7245.2022.03.008

[16] Zhang L Wu S Shao Z Guo J Wang J Xu W . Effect of non-high-density lipoprotein cholesterol on cardiovascular disease in postmenopausal women. Chin Circ J. (2024) 39:61–7.

[17] Expert Committee on Medical Nutrition Therapy for Overweight/Obesity in China . Chinese expert consensus on medical nutrition therapy for overweight/obesity (2016 edition). Chin J Diabetes. (2016) 8:525–40.

[18] Chinese Diabetes Society . Chinese guidelines for the prevention and treatment of type 2 diabetes (2020 edition). Chin J Endocrinol Metab. (2021) 37:311–98. doi: 10.2337/diacare.27.2007.s133 14693949

[19] Chinese Hypertension Guidelines Revision Committee, Hypertension Alliance, Hypertension Branch of China International Exchange and Promotive Association for Medical and Health Care, Hypertension Branch of Chinese Geriatrics Society, Hypertension Branch of Chinese Elderly Health Care Association, Chinese Stroke Association . Chinese hypertension guideline (2024 revision). Chin J Hypertens. (2024) 32:603–700. doi: 10.16439/j.issn.1673-7245.2024.07.002

[20] Joint Committee on Revision of Chinese Guidelines for Lipid Management . Chinese guidelines for lipid management (primary care edition 2024). Chin J Cardiol. (2024) 52:330–7.

[21] Huang X Li C Zeng P Ling Y Tan S Bai Z . Non-traditional lipid-inflammatory parameters estimate the risk of stroke in middle-aged and older Chinese adults: a nationwide prospective cohort study. J Adv Res. (2025). doi: 10.1016/j.jare.2025.11.029 41260379 PMC13453658

[22] Yu B Li M Yu Z Zheng T Feng X Gao A . The non-high-density lipoprotein cholesterol to high-density lipoprotein cholesterol ratio (NHHR) as a predictor of all-cause and cardiovascular mortality in US adults with diabetes or prediabetes: NHANES 1999-2018. BMC Med. (2024) 22:317. doi: 10.1186/s12916-024-03536-3 39113030 PMC11304565

[23] Mansoori A Nosrati M Dorchin M Mohammadyari F Derakhshan‐Nezhad E Ferns G . A novel index for diagnosis of type 2 diabetes mellitus: cholesterol, high density lipoprotein, and glucose (CHG) index. J Diabetes Invest. (2024) 16:309–14. doi: 10.1111/jdi.14343 39569998 PMC11786182

[24] Yu Y Zhang Y Zhu C Duan T Rao Z . Remnant cholesterol inflammatory index, calculated from residual cholesterol to C-reactive protein ratio, and stroke outcomes: a retrospective study using the National Institutes of Health Stroke Scale and modified Rankin scale. Lipids Health Dis. (2025) 24. doi: 10.1186/s12944-025-02650-2 40604981 PMC12220354

[25] Zhao E Wang R Chen Y Xie H Gao Y Dong B . Evaluation of a new TyG indicator, TyHGB, in predicting non-alcoholic fatty liver disease: evidence from two independent populations. Lipids Health Dis. (2025) 24. doi: 10.1186/s12944-025-02742-z 41131568 PMC12551267

[26] Shang W Li J Zhuo J Zhuang X Li J . C-reactive protein-triglyceride-glucose index (CTI) as a predictor of chronic liver disease in adults with macrocytic anemia. Sci Rep. (2026). doi: 10.1038/s41598-026-52488-6 42106545 PMC13347043

[27] Yang M Hu LY Liu T Yi YY Xu YH . Association of the hs-CRP-TyG index with coronary artery disease risk and angiographic severity: a retrospective comparative study with the TyG index. Front Endocrinol (Lausanne). (2026) 17:1836149. doi: 10.3389/fendo.2026.1836149 42181189 PMC13189774

[28] Huang Y Xu Y Zhang C Gu W Li Y Zhang H . Comparative performance of insulin resistance-related indices in predicting adverse cardiovascular events among individuals with NAFLD and MASLD: a multi-center cohort study. Cardiovasc Diabetol. (2026) 25. doi: 10.1186/s12933-026-03208-x 42401883 PMC13335360

[29] Tian Z Yan X Xue F Yang J Li N . Associations between the C-reactive protein-triglyceride-glucose index and its derived indices and the incidence and progression of cardiometabolic multimorbidity in participants with metabolic dysfunction-associated steatotic liver disease: a large-scale prospective cohort study. Cardiovasc Diabetol. (2026). doi: 10.1186/s12933-026-03260-7 42323654 PMC13508244

[30] Zhang L Li S Liu D Gui J Hu J Wang Q . The relationship between C-reactive protein-triglyceride-glucose index and cardiovascular disease: insights from the China health and retirement longitudinal study (CHARLS). Cardiovasc Diabetol. (2025) 24:410. doi: 10.1186/s12933-025-02960-w 41152893 PMC12560464

[31] Liu L Yu G Ji X Wang Y He H . Associations of six insulin resistance-related indices with the risk and progression of cardio-renal-metabolic multimorbidity: evidence from the UK biobank. Cardiovasc Diabetol. (2025) 24:377. doi: 10.1186/s12933-025-02928-w 41029369 PMC12487372

[32] Tian Y Zhang X Gao S Wang H . Decoding the lipid etiology of atherogenic index of plasma and gout: establishing the causal role of triglycerides through NHANES, Mendelian randomization, and network pharmacology. Cardiovasc Diabetol Endocrinol Rep. (2026) 12. doi: 10.1186/s40842-026-00309-0 42437949 PMC13362044

[33] Xie S Galimberti F Olmastroni E Luscher TF Carugo S Catapano AL . Effect of lipid-lowering therapies on C-reactive protein levels: a comprehensive meta-analysis of randomized controlled trials. Cardiovasc Res. (2024) 120:333–44. doi: 10.1093/cvr/cvae034 38373008 PMC10981526

[34] Jin Y Xu Z Zhang Y Zhang Y Wang D Cheng Y . Serum/plasma biomarkers and the progression of cardiometabolic multimorbidity: a systematic review and meta-analysis. Front Public Health. (2023) 11. doi: 10.3389/fpubh.2023.1280185 38074721 PMC10701686

[35] Khafagy R Dash S . Obesity and cardiovascular disease: the emerging role of inflammation. Front Cardiovasc Med. (2021) 8. doi: 10.3389/fcvm.2021.768119 34760952 PMC8573144

[36] Hua H Zhao J Zhou Z He S Mei S Fei X . The combined effects of cardiometabolic index and high sensitivity C-reactive protein on cardiometabolic multimorbidity risk: a prospective analysis based on CHARLS. Lipids Health Dis. (2026) 25. doi: 10.1186/s12944-026-02904-7 41731484 PMC13037248

[37] Libby P Ridker PM Hansson GK . Inflammation in atherosclerosis. J Am Coll Cardiol. (2009) 54:2129–38. doi: 10.1038/nature01323 19942084 PMC2834169

[38] Dang K Wang X Hu J Zhang Y Cheng L Qi X . The association between triglyceride-glucose index and its combination with obesity indicators and cardiovascular disease: NHANES 2003–2018. Cardiovasc Diabetol. (2024) 23. doi: 10.1186/s12933-023-02115-9 38184598 PMC10771672

[39] Chen X Wang C Huang Q Luo Y Wu Z Chen J . Associations of cumulative exposure and dynamic trajectories of the triglyceride–total cholesterol–body weight index with new-onset cardiometabolic multimorbidity in middle-aged and older Chinese adults: evidence from a nationwide prospective cohort study. Cardiovasc Diabetol. (2026) 25. doi: 10.1186/s12933-026-03155-7 41906174 PMC13151372

[40] Cui C Liu L Qi Y Han N Xu H Wang Z . Joint association of TyG index and high sensitivity C-reactive protein with cardiovascular disease: a national cohort study. Cardiovasc Diabetol. (2024) 23. doi: 10.1186/s12933-024-02244-9 38715129 PMC11077847

[41] Monteiro R Azevedo I . Chronic inflammation in obesity and the metabolic syndrome. Mediators Inflammation. (2010) 2010. doi: 10.1155/2010/289645 20706689 PMC2913796

[42] Chen K Li F Li J Cai H Strom S Bisello A . Induction of leptin resistance through direct interaction of C-reactive protein with leptin. Nat Med. (2006) 12:425–32. doi: 10.1038/nm1372 16582918

[43] Yousuf O Mohanty BD Martin SS Joshi PH Blaha MJ Nasir K . High-sensitivity C-reactive protein and cardiovascular disease. J Am Coll Cardiol. (2013) 62:397–403. doi: 10.1016/j.jacc.2013.05.016 23727085

[44] Olefsky JM Glass CK . Macrophages, inflammation, and insulin resistance. Annu Rev Physiol. (2010) 72:219–46. doi: 10.1146/annurev-physiol-021909-135846 20148674

[45] Duran EK Cook NR Aday AW Buring JE Ridker PM Pradhan AD . Unsupervised learning analysis of triglycerides, inflammation, cholesterol, and the risks of incident cardiovascular disease and type 2 diabetes in the women's health study. J Am Heart Assoc. (2025) 14. doi: 10.1161/jaha.123.039381 40913283 PMC12554421

[46] LiuLi A Zhang T Feng Z . Adipose tissue distribution in metabolic disease: depot-specific biology, clinical assessment, and therapeutic remodeling. Front Endocrinol (Lausanne). (2026) 17:1822659. doi: 10.3389/fendo.2026.1822659 42422426 PMC13341497

[47] Hu J Shang C Huang Y Sun C Zhang J . Insulin resistance-related indices, genetic risk, and the risk of cardiovascular disease in individuals with preclinical or clinical obesity: a large prospective cohort study in the UK biobank. Cardiovasc Diabetol. (2025) 24. doi: 10.1186/s12933-025-02961-9 41139206 PMC12554247

[48] Jankauskas SS Komici K Varzideh F Aversa LS Kansakar U D'Onghia ML . Cardiovascular-kidney-metabolic syndrome: a comprehensive review of pathophysiology, epidemiology, diagnosis, and management. Cardiovasc Diabetol. (2026) 25. doi: 10.1186/s12933-026-03177-1 42057054 PMC13274062

[49] Lian PA Xie F Zhang W Cheng S Zhao YF Li L . Association of the atherogenic index of plasma and high-sensitivity C-reactive protein with incident cardiovascular disease: evidence from a national cohort of middle-aged and older Chinese adults. Front Endocrinol (Lausanne). (2025) 16:1618157. doi: 10.3389/fendo.2025.1618157 40842496 PMC12364642

[50] Lu A Xu Q . Association between the triglyceride-high-density lipoprotein-glucose-body index and new-onset stroke risk: a prospective cohort study. Lipids Health Dis. (2026). doi: 10.1186/s12944-026-02953-y 42002766 PMC13224718

[51] Hou X Wang G Gao Y . The association between triglyceride glucose-frailty index and cardiometabolic multimorbidity among Chinese middle-aged and older adults: a national prospective cohort study. Cardiovasc Diabetol. (2026). doi: 10.1186/s12933-026-03191-3 42071205 PMC13471258

[52] Wang Y Wang H Liu X Zhang J . Improving cardiometabolic multimorbidity prediction with a composite obesity-TyG index: a study of middle-aged and older adults in CHARLS. Arch Public Health. (2025) 83. doi: 10.1186/s13690-025-01762-6 41219986 PMC12606983

[53] Santulli G . Epidemiology of cardiovascular disease and diabetes in 2026: expanding prevalence, escalating costs, and the limits of contemporary prevention. Cardiovasc Diabetol Endocrinol Rep. (2026) 12. doi: 10.1186/s40842-026-00283-7 41918034 PMC13041046

[54] Mohamed R Hendawy LMA Farghaly EEM Saleh A . Clinical characteristics and outcomes of diabetic ketoacidosis in patients with type 2 diabetes during acute systemic stress. Cardiovasc Diabetol Endocrinol Rep. (2026) 12. doi: 10.1186/s40842-026-00293-5 42252461 PMC13244619

