# Supplementary material for: Cumulative lipid–inflammatory indices and the risk of cardiometabolic multimorbidity: a prospective cohort study
Source: Front Endocrinol (Lausanne). 2026 Aug 10;17:1909025. doi: 10.3389/fendo.2026.1909025 (PMC13500255; doi:10.3389/fendo.2026.1909025)
Supplement: Supplementary file 1 [file DataSheet1.pdf]

Table S1 Missing Covariates

Covariate	Number of Missing Values	Percentage of Missing Values
BMI(kg/m^2)	42	0.17%
WC(cm)	69	0.27%
SBP(mmHg)	150	0.59%
DBP(mmHg)	150	0.59%
HR(bpm)	987	3.89%
UA(μmol/L)	61	0.24%
Exercise(%)	30	0.12%
Smoke(%)	27	0.11%
Drink(%)	33	0.13%

Abbreviations: BMI body mass index, WC waist circumference, SBP systolic blood pressure, DBP diastolic blood pressure, HR heart rate,UA uric acid

Table S2 Comparison of baseline characteristics between included and excluded participants

Variable	Participants with missing biomarkers (n=15,948)	Final analytical sample (n=25,376)
Age(years)	50.57±12.00	52.07±11.68
Male(%)	11,785(73.90)	19,135(75.41)
BMI(kg/m^2)	24.22±3.30	24.60±3.24
WC(cm)	86.43±10.11	87.71±10.21
SBP(mmHg)	127.42±18.05	129.55±18.88
DBP(mmHg)	83.33±10.70	83.95±10.79
FBG(mg/dL)	93.44±10.79	94.62±10.65
HR(bpm)	72.70±10.30	72.84±9.92
TG(mg/dL)	108.02(81.46-150.53)	111.57(77.03-168.24)
TC(mg/dL)	185.63±49.56	195.56±46.77
HDL-C(mg/dL)	62.64±22.93	59.87±16.71
LDL-C(mg/dL)	98.73±44.84	102.00±30.32
UA(μmol/L)	277.02±86.81	300.25±87.68
CR(μmol/L)	80.14±26.07	79.30±21.34
hs-CRP(mg/L)	0.62(0.00-1.30)	1.19(0.60-2.42)
Exercise(%)	10,022(63.44)	17,225(67.88)
Hypertension(%)	8,321(52.18)	13,577(53.50)
Hyperlipidemia(%)	9,654(60.53)	16,883(66.53)
Antihypertensive agents(%)	3,041(19.07)	5,526(21.78)
Lipid-lowering drugs(%)	901(5.65)	1,635(6.44)
Smoke(%)	5,330(33.74)	8,777(34.59)
Drink(%)	5,494(34.78)	9,353(36.86)

Data are presented as mean (SD) or median (interquartile range) for continuous variables and n (%) for categorical variables

BMI body mass index, *WC* waist circumference, *SBP* systolic blood pressure, *DBP* diastolic blood pressure, *FBG* fasting blood glucose, *HR* heart rate, *TG* triglyceride, *TC* total cholesterol, *HDL-C* high density lipoprotein cholesterol, *LDL-C* low density lipoprotein cholesterol, *UA* uric acid, *CR* creatinine, *hs-CRP h*igh-sensitivity C-reactive protein

Table S3 HR for risk of CMM subtypes according to tertiles of the nine cumulative lipid-inflammatory indices

	Event(%)	HR(95%CI)		
		Model I	Model II	Model III
**IHD**				
**cumAIP_CRP**				
Per 1 SD		1.25(1.20-1.31)	1.23(1.17-1.29)	1.04(0.98-1.10)
T1	172(2.03)	ref	ref	ref
T2	237(2.80)	1.39(1.14-1.69)	1.32(1.08-1.61)	0.94(0.77-1.15)
T3	396(4.68)	2.36(1.97-2.82)	2.16(1.80-2.58)	1.13(0.93-1.38)
P for trend		<0.001	<0.001	0.098
**cumCRI_I_CRP**				
Per 1 SD		1.26(1.21-1.30)	1.23(1.18-1.28)	1.10(1.04-1.16)
T1	148(1.75)	ref	ref	ref
T2	248(2.93)	1.70(1.39-2.09)	1.52(1.24-1.87)	1.17(0.95-1.44)
T3	409(4.84)	2.90(2.40-3.49)	2.41(2.00-2.92)	1.43(1.17-1.74)
P for trend		<0.001	<0.001	<0.001
**cumCRI_II_CRP**				
Per 1 SD		1.23(1.19-1.26)	1.21(1.17-1.25)	1.11(1.05-1.17)
T1	155(1.83)	ref	ref	ref
T2	236(2.79)	1.55(1.26-1.89)	1.38(1.13-1.69)	1.03(0.84-1.27)
T3	414(4.89)	2.80(2.33-3.37)	2.31(1.91-2.78)	1.35(1.11-1.64)
P for trend		<0.001	<0.001	<0.001
**cumLCI_CRP**				
Per 1 SD		1.07(1.05-1.09)	1.07(1.05-1.09)	1.03(0.99-1.07)
T1	130(1.54)	ref	ref	ref
T2	247(2.92)	1.93(1.56-2.38)	1.70(1.37-2.10)	1.15(0.92-1.43)
T3	428(5.06)	3.42(2.81-4.16)	2.97(2.44-3.61)	1.48(1.19-1.83)
P for trend		<0.001	<0.001	<0.001
**cumNHHR_CRP**				
Per 1 SD		1.21(1.17-1.24)	1.19(1.15-1.23)	1.09(1.04-1.15)
T1	142(1.68)	ref	ref	ref
T2	246(2.91)	1.76(1.43-2.16)	1.57(1.27-1.93)	1.17(0.94-1.44)
T3	417(4.93)	3.08(2.54-3.72)	2.56(2.12-3.11)	1.50(1.23-1.83)
P for trend		<0.001	<0.001	<0.001
**cumCHG_CRP**				
Per 1 SD		1.35(1.27-1.42)	1.26(1.19-1.33)	1.08(1.02-1.15)
T1	164(1.94)	ref	ref	ref
T2	259(3.06)	1.61(1.32-1.96)	1.44(1.18-1.75)	1.10(0.90-1.34)
T3	382(4.52)	2.43(2.03-2.92)	2.04(1.69-2.45)	1.24(1.02-1.50)
P for trend		<0.001	<0.001	0.024
**cumLnRCII**				
Per 1 SD		1.61(1.49-1.73)	1.49(1.38-1.61)	1.16(1.07-1.25)
T1	146(1.73)	ref	ref	ref
T2	266(3.14)	1.79(1.46-2.19)	1.64(1.34-2.01)	1.27(1.03-1.55)
T3	393(4.65)	2.85(2.36-3.45)	2.43(2.01-2.95)	1.41(1.16-1.72)
P for trend		<0.001	<0.001	<0.001

**cumTyHGB_CRP**				
Per 1 SD		1.35(1.28-1.43)	1.26(1.19-1.33)	1.07(1.01-1.14)
T1	157(1.86)	ref	ref	ref
T2	251(2.97)	1.63(1.33-1.99)	1.45(1.19-1.77)	1.09(0.89-1.33)
T3	397(4.69)	2.65(2.20-3.19)	2.19(1.82-2.64)	1.27(1.05-1.54)
P for trend		<0.001	<0.001	0.010
**cumCTI**				
Per 1 SD		1.43(1.34-1.52)	1.25(1.16-1.34)	1.01(0.93-1.09)
T1	152(1.80)	ref	ref	ref
T2	294(3.48)	2.01(1.65-2.45)	1.62(1.33-1.98)	1.26(1.03-1.54)
T3	359(4.24)	2.56(2.12-3.10)	1.77(1.45-2.16)	1.11(0.90-1.37)
P for trend		<0.001	<0.001	0.602
**Stroke**				
**cumAIP_CRP**				
Per 1 SD		1.16(1.12-1.21)	1.13(1.08-1.17)	1.04(0.99-1.09)
T1	441(5.21)	ref	ref	ref
T2	581(6.87)	1.34(1.18-1.51)	1.29(1.14-1.46)	1.13(0.99-1.28)
T3	688(8.13)	1.62(1.44-1.82)	1.47(1.31-1.66)	1.16(1.02-1.33)
P for trend		<0.001	<0.001	0.036
**cumCRI_I_CRP**				
Per 1 SD		1.17(1.13-1.21)	1.11(1.06-1.15)	1.05(1.00-1.10)
T1	443(5.24)	ref	ref	ref
T2	568(6.71)	1.31(1.16-1.48)	1.19(1.05-1.34)	1.05(0.92-1.19)
T3	699(8.26)	1.61(1.43-1.81)	1.37(1.21-1.54)	1.12(0.98-1.27)
P for trend		<0.001	<0.001	0.087
**cumCRI_II_CRP**				
Per 1 SD		1.15(1.11-1.18)	1.09(1.05-1.13)	1.03(0.98-1.08)
T1	427(5.05)	ref	ref	ref
T2	606(7.16)	1.46(1.29-1.65)	1.30(1.15-1.47)	1.18(1.04-1.34)
T3	677(8.00)	1.69(1.50-1.91)	1.35(1.19-1.52)	1.12(0.98-1.27)
P for trend		<0.001	<0.001	0.141
**cumLCI_CRP**				
Per 1 SD		1.10(1.07-1.12)	1.09(1.06-1.12)	1.05(1.01-1.09)
T1	425(5.02)	ref	ref	ref
T2	611(7.22)	1.48(1.30-1.67)	1.30(1.15-1.47)	1.12(0.98-1.27)
T3	674(7.97)	1.67(1.48-1.88)	1.43(1.27-1.62)	1.07(0.93-1.23)
P for trend		<0.001	<0.001	0.423
**cumNHHR_CRP**				
Per 1 SD		1.14(1.11-1.17)	1.10(1.06-1.14)	1.04(1.00-1.09)
T1	449(5.31)	ref	ref	ref
T2	567(6.70)	1.29(1.14-1.46)	1.15(1.01-1.30)	1.02(0.90-1.16)
T3	694(8.20)	1.64(1.45-1.84)	1.32(1.17-1.49)	1.08(0.95-1.23)
P for trend		<0.001	<0.001	0.197
**cumCHG_CRP**				
Per 1 SD		1.21(1.17-1.27)	1.11(1.07-1.16)	1.05(1.01-1.10)
T1	453(5.36)	ref	ref	ref
T2	574(6.79)	1.30(1.15-1.47)	1.16(1.02-1.31)	1.05(0.93-1.19)
T3	683(8.07)	1.59(1.42-1.80)	1.30(1.15-1.46)	1.11(0.98-1.25)

P for trend		<0.001	<0.001	0.106
**cumLnRCII**				
Per 1 SD		1.26(1.19-1.32)	1.14(1.09-1.20)	1.04(0.99-1.10)
T1	457(5.40)	ref	ref	ref
T2	541(6.40)	1.20(1.06-1.36)	1.10(0.97-1.25)	0.99(0.87-1.12)
T3	712(8.42)	1.63(1.45-1.84)	1.36(1.21-1.53)	1.10(0.97-1.25)
P for trend		<0.001	<0.001	0.097
**cumTyHGB_CRP**				
Per 1 SD		1.23(1.18-1.28)	1.12(1.08-1.17)	1.06(1.01-1.10)
T1	453(5.36)	ref	ref	ref
T2	572(6.76)	1.30(1.15-1.47)	1.15(1.01-1.30)	1.03(0.91-1.17)
T3	685(8.10)	1.60(1.42-1.80)	1.29(1.14-1.45)	1.07(0.95-1.22)
P for trend		<0.001	<0.001	0.267
**cumCTI**				
Per 1 SD		1.36(1.30-1.42)	1.16(1.11-1.22)	1.07(1.01-1.13)
T1	393(4.65)	ref	ref	ref
T2	576(6.81)	1.56(1.37-1.77)	1.23(1.08-1.41)	1.09(0.95-1.24)
T3	741(8.76)	2.18(1.93-2.47)	1.44(1.26-1.64)	1.18(1.02-1.35)
P for trend		<0.001	<0.001	0.022
**T2D**				
**cumAIP_CRP**				
Per 1 SD		1.31(1.28-1.33)	1.30(1.28-1.33)	1.20(1.17-1.23)
T1	547(6.47)	ref	ref	ref
T2	1020(12.06)	1.73(1.57-1.90)	1.71(1.55-1.88)	1.34(1.21-1.48)
T3	1582(18.70)	2.25(2.06-2.47)	2.24(2.03-2.47)	1.53(1.38-1.70)
P for trend		<0.001	<0.001	<0.001
**cumCRI_I_CRP**				
Per 1 SD		1.22(1.20-1.25)	1.22(1.19-1.24)	1.13(1.10-1.16)
T1	669(7.91)	ref	ref	ref
T2	1055(12.47)	1.63(1.48-1.80)	1.60(1.45-1.76)	1.30(1.18-1.43)
T3	1425(16.85)	2.31(2.10-2.53)	2.25(2.05-2.47)	1.56(1.42-1.72)
P for trend		<0.001	<0.001	<0.001
**cumCRI_II_CRP**				
Per 1 SD		1.19(1.17-1.21)	1.19(1.17-1.21)	1.12(1.09-1.15)
T1	666(7.87)	ref	ref	ref
T2	1056(12.48)	1.64(1.49-1.81)	1.61(1.46-1.77)	1.32(1.20-1.46)
T3	1427(16.87)	2.32(2.12-2.54)	2.26(2.06-2.49)	1.56(1.42-1.73)
P for trend		<0.001	<0.001	<0.001
**cumLCI_CRP**				
Per 1 SD		1.07(1.06-1.08)	1.07(1.06-1.08)	1.06(1.04-1.07)
T1	559(6.61)	ref	ref	ref
T2	986(11.66)	1.82(1.64-2.02)	1.78(1.60-1.97)	1.41(1.27-1.58)
T3	1604(18.96)	3.13(2.84-3.45)	3.05(2.77-3.36)	2.03(1.82-2.26)
P for trend		<0.001	<0.001	<0.001
**cumNHHR_CRP**				
Per 1 SD		1.18(1.16-1.20)	1.18(1.16-1.20)	1.12(1.09-1.15)
T1	661(7.82)	ref	ref	ref
T2	1059(12.52)	1.65(1.50-1.82)	1.62(1.47-1.79)	1.30(1.18-1.44)

T3	1429(16.89)	2.34(2.13-2.56)	2.29(2.08-2.51)	1.56(1.42-1.72)
P for trend		<0.001	<0.001	<0.001
**cumCHG_CRP**				
Per 1 SD		1.26(1.23-1.30)	1.26(1.22-1.30)	1.13(1.09-1.17)
T1	732(8.65)	ref	ref	ref
T2	1074(12.70)	1.52(1.38-1.67)	1.49(1.35-1.63)	1.23(1.12-1.36)
T3	1343(15.88)	1.97(1.80-2.15)	1.92(1.75-2.10)	1.39(1.27-1.53)
P for trend		<0.001	<0.001	<0.001
**cumLnRCII**				
Per 1 SD		1.42(1.37-1.47)	1.41(1.36-1.47)	1.20(1.15-1.25)
T1	677(8.00)	ref	ref	ref
T2	1061(12.54)	1.62(1.47-1.78)	1.59(1.44-1.75)	1.29(1.17-1.43)
T3	1411(16.68)	2.24(2.04-2.45)	2.18(1.99-2.40)	1.51(1.37-1.67)
P for trend		<0.001	<0.001	<0.001
**cumTyHGB_CRP**				
Per 1 SD		1.37(1.33-1.41)	1.36(1.33-1.40)	1.23(1.19-1.26)
T1	633(7.48)	ref	ref	ref
T2	1044(12.34)	1.71(1.55-1.89)	1.68(1.52-1.86)	1.39(1.25-1.53)
T3	1472(17.40)	2.54(2.31-2.79)	2.49(2.26-2.73)	1.79(1.62-1.97)
P for trend		<0.001	<0.001	<0.001
**cumCTI**				
Per 1 SD		1.37(1.33-1.42)	1.37(1.33-1.42)	1.19(1.14-1.24)
T1	668(7.90)	ref	ref	ref
T2	1104(13.05)	1.73(1.57-1.90)	1.71(1.55-1.88)	1.34(1.21-1.48)
T3	1377(16.28)	2.25(2.06-2.47)	2.24(2.03-2.47)	1.53(1.38-1.70)
P for trend		<0.001	<0.001	<0.001

Model I: non-adjusted

Model II: adjusted for age, sex

Model III: adjusted for Model II+BMI, SBP, DBP, WC, HR, CR, UA, hypertension, hyperlipidemia, exercise, drink, smoke, and medication use (antihypertensive agents, lipid-lowering drugs)

Table S4 Two-piecewise Cox regression for CMM subtypes stratified by RCS-derived breakpoints

Variable	HR(95%CI)	*P* value
**IHD**		
The inflection point of cumAIP_CRP	3.32	
<3.32	1.14(1.03,1.25)	0.012
>3.32	0.37(0.14,1.00)	0.051
P for likelihood test	0.003	
The inflection point of cumCRI_I_CRP	4.65	
<4.65	1.12(1.06,1.18)	<0.001
>4.65	0.99(0.94,1.04)	0.719
P for likelihood test	0.005	
The inflection point of cumCRI_II_CRP	2.09	
<2.09	1.27(1.13,1.44)	<0.001
>2.09	1.03(0.96,1.10)	0.458
P for likelihood test	0.008	
The inflection point of cumLCI_CRP	49557.21	
<49557.21	1.22(1.15,1.30)	<0.001
>49557.21	1.01(0.97,1.06)	0.643
P for likelihood test	<0.001	
The inflection point of cumNHHR_CRP	3.64	
<3.64	1.17(1.09,1.25)	<0.001
>3.64	0.99(0.93,1.05)	0.654
P for likelihood test	0.001	
**Stroke**		
The inflection point of cumLCI_CRP	6176.419	
<6176.419	0.92 (0.86,0.99)	0.013
>6176.419	1.24 (1.02,1.51)	0.035
P for likelihood test	0.011	
**T2D**		
The inflection point of cumAIP_CRP	0.39	
<0.39	3.64(2.78,4.76)	<0.001
>0.39	1.20(1.15,1.25)	<0.001
P for likelihood test	<0.001	
The inflection point of cumCRI_I_CRP	1.43	
<1.43	1.52(1.32,1.74)	<0.001
>1.43	1.04(1.03,1.06)	<0.001
P for likelihood test	<0.001	
The inflection point of cumCRI_II_CRP	0.61	
<0.61	2.33(1.66,3.26)	<0.001
>0.61	1.08(1.06,1.11)	<0.001

P for likelihood test	<0.001	
The inflection point of cumLCI_CRP	41486.37	
<41486.37	1.71 (1.54, 1.90)	<0.001
>41486.37	1.13 (1.04, 1.23)	0.002
P for likelihood test	<0.001	
The inflection point of cumNHHR_CRP	0.93	
<0.93	1.94(1.57,2.41)	<0.001
>0.93	1.05(1.04,1.07)	<0.001
P for likelihood test	<0.001	
The inflection point of cumCHG_CRP	7.60	
<7.60	1.07(1.04,1.09)	<0.001
>7.60	1.01(1.00,1.01)	0.005
P for likelihood test	<0.001	
The inflection point of cumTyHGB_CRP	40.20	
<40.20	1.02(1.01,1.02)	<0.001
>40.20	1.00(1.00,1.00)	<0.001
P for likelihood test		
The inflection point of cumCTI	32.21	
<32.21	1.12(1.08,1.16)	<0.001
>32.21	1.03(1.02,1.04)	<0.001
P for likelihood test	<0.001	

Models were adjusted for age, sex, BMI, SBP, DBP, WC, HR, CR, UA, hypertension, hyperlipidemia, exercise, drink, smoke, and medication use (antihypertensive agents,lipid-lowering drugs)

Table S5 Competing Risk Model for Mortality

Variable	HR(95%CI)		
	**Model I**	**Model II**	**Model III**
**cumAIP_CRP**			
T1	ref	ref	ref
T2	1.88(1.42-2.49)	1.81(1.37-2.40)	1.30(0.97-1.74)
T3	3.61(2.79-4.66)	3.37(2.60-4.35)	1.87(1.41-2.49)
P for trend	<0.001	<0.001	<0.001
**cumCRI_I_CRP**			
T1	ref	ref	ref
T2	1.81(1.38-2.38)	1.68(1.28-2.21)	1.27(0.96-1.67)
T3	3.26(2.54-4.19)	2.89(2.24-3.72)	1.74(1.33-2.26)
P for trend	<0.001	<0.001	<0.001
**cumCRI_II_CRP**			
T1	ref	ref	ref
T2	1.95(1.48-2.57)	1.81(1.38-2.39)	1.38(1.04-1.83)
T3	3.38(2.62-4.36)	2.97(2.29-3.84)	1.76(1.34-2.31)
P for trend	<0.001	<0.001	<0.001
**cumLCI_CRP**			
T1	ref	ref	ref
T2	2.38(1.77-3.22)	2.18(1.62-2.95)	1.56(1.14-2.12)
T3	4.63(3.51-6.10)	4.18(3.16-5.52)	2.25(1.65-3.05)
P for trend	<0.001	<0.001	<0.001
**cumNHHR_CRP**			
T1	ref	ref	ref
T2	1.88(1.43-2.49)	1.75(1.32-2.31)	1.29(0.97-1.71)
T3	3.43(2.66-4.43)	3.04(2.35-3.93)	1.80(1.37-2.36)
P for trend	<0.001	<0.001	<0.001
**cumCHG_CRP**			
T1	ref	ref	ref
T2	1.83(1.41-2.38)	1.70(1.30-2.21)	1.29(0.99-1.68)
T3	2.74(2.14-3.51)	2.43(1.89-3.12)	1.52(1.17-1.97)
P for trend	<0.001	<0.001	0.001
**cumLnRCII**			
T1	ref	ref	ref
T2	1.70(1.29-2.22)	1.60(1.22-2.09)	1.20(0.91-1.59)
T3	3.13(2.44-4.00)	2.80(2.18-3.59)	1.67(1.28-2.19)
P for trend	<0.001	<0.001	<0.001
**cumTyHGB_CRP**			
T1	ref	ref	ref
T2	2.00(1.52-2.64)	1.85(1.40-2.44)	1.38(1.04-1.82)
T3	3.39(2.62-4.38)	2.99(2.31-3.87)	1.80(1.38-2.36)
P for trend	<0.001	<0.001	<0.001
**cumCTI**			
T1	ref	ref	ref
T2	2.13(1.62-2.79)	1.89(1.43-2.49)	1.36(1.03-1.81)
T3	3.40(2.63-4.40)	2.79(2.12-3.66)	1.68(1.26-2.24)

P for trend	<0.001	<0.001	<0.001

Model I: non-adjusted

Model II: adjusted for age, sex

Model III: adjusted for Model II+BMI, SBP, DBP, WC, HR, CR, UA, hypertension, hyperlipidemia, exercise, drink, smoke, and medication use (antihypertensive agents, lipid-lowering drugs)

Table S6 Sensitivity Analysis

	HR(95%CI)		
	**Model I**	**Model II**	**Model III**
**1.Exclude participants who experienced CMM event during the first two years of follow-up(n=25,238)**			
**cumAIP_CRP**			
T1	ref	ref	ref
T2	1.91(1.44-2.53)	1.82(1.38-2.42)	1.32(0.99-1.77)
T3	3.69(2.85-4.78)	3.41(2.63-4.41)	1.92(1.45-2.54)
P for trend	<0.001	<0.001	<0.001
**cumCRI_I_CRP**			
T1	ref	ref	ref
T2	1.82(1.39-2.40)	1.66(1.26-2.19)	1.26(0.96-1.66)
T3	3.38(2.63-4.35)	2.93(2.27-3.77)	1.77(1.36-2.31)
P for trend	<0.001	<0.001	<0.001
**cumCRI_II_CRP**			
T1	ref	ref	ref
T2	1.97(1.50-2.60)	1.80(1.37-2.37)	1.38(1.05-1.83)
T3	3.49(2.70-4.50)	2.99(2.31-3.87)	1.78(1.36-2.33)
P for trend	<0.001	<0.001	<0.001
**cumLCI_CRP**			
T1	ref	ref	ref
T2	2.39(1.77-3.22)	2.15(1.59-2.90)	1.54(1.13-2.10)
T3	4.72(3.57-6.22)	4.18(3.17-5.52)	2.27(1.67-3.07)
P for trend	<0.001	<0.001	<0.001
**cumNHHR_CRP**			
T1	ref	ref	ref
T2	1.89(1.43-2.49)	1.72(1.30-2.27)	1.27(0.96-1.6)
T3	3.55(2.75-4.58)	3.07(2.38-3.97)	1.82(1.39-2.39)
P for trend	<0.001	<0.001	<0.001
**cumCHG_CRP**			
T1	ref	ref	ref
T2	1.84(1.42-2.40)	1.69(1.30-2.19)	1.30(1.00-1.70)
T3	2.84(2.22-3.64)	2.47(1.93-3.17)	1.56(1.21-2.02)
P for trend	<0.001	<0.001	<0.001
**cumLnRCII**			
T1	ref	ref	ref
T2	1.70(1.30-2.23)	1.58(1.21-2.08)	1.19(0.91-1.57)
T3	3.21(2.51-4.11)	2.81(2.19-3.61)	1.69(1.29-2.20)
P for trend	<0.001	<0.001	<0.001
**cumTyHGB_CRP**			
T1	ref	ref	ref
T2	2.02(1.53-2.66)	1.84(1.40-2.43)	1.38(1.04-1.82)
T3	3.52(2.72-4.54)	3.04(2.34-3.93)	1.85(1.41-2.41)
P for trend	<0.001	<0.001	<0.001
**cumCTI**			
T1	ref	ref	ref
T2	2.17(1.66-2.85)	1.87(1.42-2.46)	1.37(1.04-1.81)
T3	3.59(2.77-4.64)	2.78(2.13-3.63)	1.69(1.28-2.24)
P for trend	<0.001	<0.001	<0.001

**2.Exclude participants who toke lipid-lowering drugs(n=23,741)**			
**cumAIP_CRP**			
T1	ref	ref	ref
T2	1.81(1.29-2.55)	1.73(1.23-2.44)	1.37(0.96-1.94)
T3	3.18(2.32-4.35)	2.93(2.14-4.02)	1.91(1.35-2.70)
P for trend	<0.001	<0.001	<0.001
**cumCRI_I_CRP**			
T1	ref	ref	ref
T2	1.65(1.19-2.30)	1.51(1.08-2.10)	1.23(0.88-1.71)
T3	2.89(2.13-3.93)	2.50(1.84-3.41)	1.76(1.27-2.43)
P for trend	<0.001	<0.001	<0.001
**cumCRI_II_CRP**			
T1	ref	ref	ref
T2	1.88(1.35-2.63)	1.72(1.23-2.40)	1.42(1.01-1.99)
T3	3.11(2.27-4.26)	2.66(1.94-3.66)	1.87(1.34-2.61)
P for trend	<0.001	<0.001	<0.001
**cumLCI_CRP**			
T1	ref	ref	ref
T2	2.23(1.56-3.19)	2.00(1.40-2.87)	1.59(1.10-2.31)
T3	4.02(2.88-5.62)	3.57(2.55-4.99)	2.38(1.64-3.46)
P for trend	<0.001	<0.001	<0.001
**cumNHHR_CRP**			
T1	ref	ref	ref
T2	1.68(1.21-2.35)	1.53(1.09-2.14)	1.23(0.88-1.73)
T3	2.97(2.18-4.04)	2.56(1.88-3.50)	1.78(1.28-2.47)
P for trend	<0.001	<0.001	<0.001
**cumCHG_CRP**			
T1	ref	ref	ref
T2	1.65(1.19-2.28)	1.50(1.09-2.08)	1.25(0.90-1.73)
T3	2.59(1.91-3.51)	2.25(1.66-3.06)	1.67(1.22-2.29)
P for trend	<0.001	<0.001	<0.001
**cumLnRCII**			
T1	ref	ref	ref
T2	1.67(1.20-2.32)	1.55(1.11-2.16)	1.27(0.91-1.78)
T3	2.81(2.07-3.82)	2.45(1.80-3.34)	1.73(1.25-2.40)
P for trend	<0.001	<0.001	<0.001
**cumTyHGB_CRP**			
T1	ref	ref	ref
T2	1.94(1.39-2.73)	1.77(1.26-2.48)	1.45(1.03-2.05)
T3	3.15(2.29-4.32)	2.71(1.97-3.74)	1.96(1.41-2.73)
P for trend	<0.001	<0.001	<0.001
**cumCTI**			
T1	ref	ref	ref
T2	2.47(1.75-3.48)	2.12(1.50-3.00)	1.64(1.15-2.34)
T3	3.70(2.66-5.16)	2.86(2.02-4.04)	1.93(1.34-2.78)
P for trend	<0.001	<0.001	<0.001
**3.Exclude participants who developed cancer during follow-up(n=24,210)**			
**cumAIP_CRP**			
T1	ref	ref	ref

T2	1.85(1.39-2.47)	1.77(1.33-2.36)	1.28(0.96-1.71)
T3	3.60(2.78-4.67)	3.32(2.56-4.31)	1.86(1.40-2.47)
P for trend	<0.001	<0.001	<0.001
**cumCRI_I_CRP**			
T1	ref	ref	ref
T2	1.86(1.41-2.46)	1.70(1.29-2.25)	1.30(0.98-1.71)
T3	3.39(2.62-4.38)	2.93(2.26-3.79)	1.78(1.36-2.33)
P for trend	<0.001	<0.001	<0.001
**cumCRI_II_CRP**			
T1	ref	ref	ref
T2	2.00(1.51-2.65)	1.83(1.38-2.42)	1.40(1.05-1.85)
T3	3.51(2.71-4.55)	3.00(2.31-3.90)	1.80(1.36-2.36)
P for trend	<0.001	<0.001	<0.001
**cumLCI_CRP**			
T1	ref	ref	ref
T2	2.48(1.83-3.37)	2.23(1.64-3.03)	1.60(1.17-2.19)
T3	4.76(3.58-6.33)	4.21(3.17-5.60)	2.28(1.67-3.12)
P for trend	<0.001	<0.001	<0.001
**cumNHHR_CRP**			
T1	ref	ref	ref
T2	1.93(1.46-2.56)	1.76(1.33-2.33)	1.30(0.98-1.73)
T3	3.57(2.75-4.63)	3.08(2.37-4.00)	1.84(1.40-2.41)
P for trend	<0.001	<0.001	<0.001
**cumCHG_CRP**			
T1	ref	ref	ref
T2	1.87(1.43-2.44)	1.71(1.31-2.23)	1.33(1.01-1.74)
T3	2.84(2.21-3.65)	2.46(1.91-3.17)	1.57(1.21-2.04)
P for trend	<0.001	<0.001	<0.001
**cumLnRCII**			
T1	ref	ref	ref
T2	1.68(1.28-2.22)	1.56(1.19-2.06)	1.18(0.90-1.56)
T3	3.19(2.48-4.10)	2.79(2.16-3.59)	1.67(1.28-2.19)
P for trend	<0.001	<0.001	<0.001
**cumTyHGB_CRP**			
T1	ref	ref	ref
T2	2.05(1.55-2.71)	1.87(1.41-2.47)	1.40(1.06-1.87)
T3	3.54(2.73-4.60)	3.05(2.34-3.98)	1.87(1.42-2.46)
P for trend	<0.001	<0.001	<0.001
**cumCTI**			
T1	ref	ref	ref
T2	2.28(1.73-3.02)	1.96(1.48-2.60)	1.44(1.08-1.92)
T3	3.73(2.86-4.86)	2.88(2.18-3.79)	1.77(1.32-2.36)
P for trend	<0.001	<0.001	<0.001

Model I: non-adjusted

Model II: adjusted for age, sex

Model III: adjusted for Model II+BMI, SBP, DBP, WC, HR, CR, UA, hypertension, hyperlipidemia, exercise, drink, smoke, and medication use (antihypertensive agents, lipid-lowering drugs)

Fig.S1 Dose-response relationships between nine cumulative lipid-inflammatory indices and stroke risk

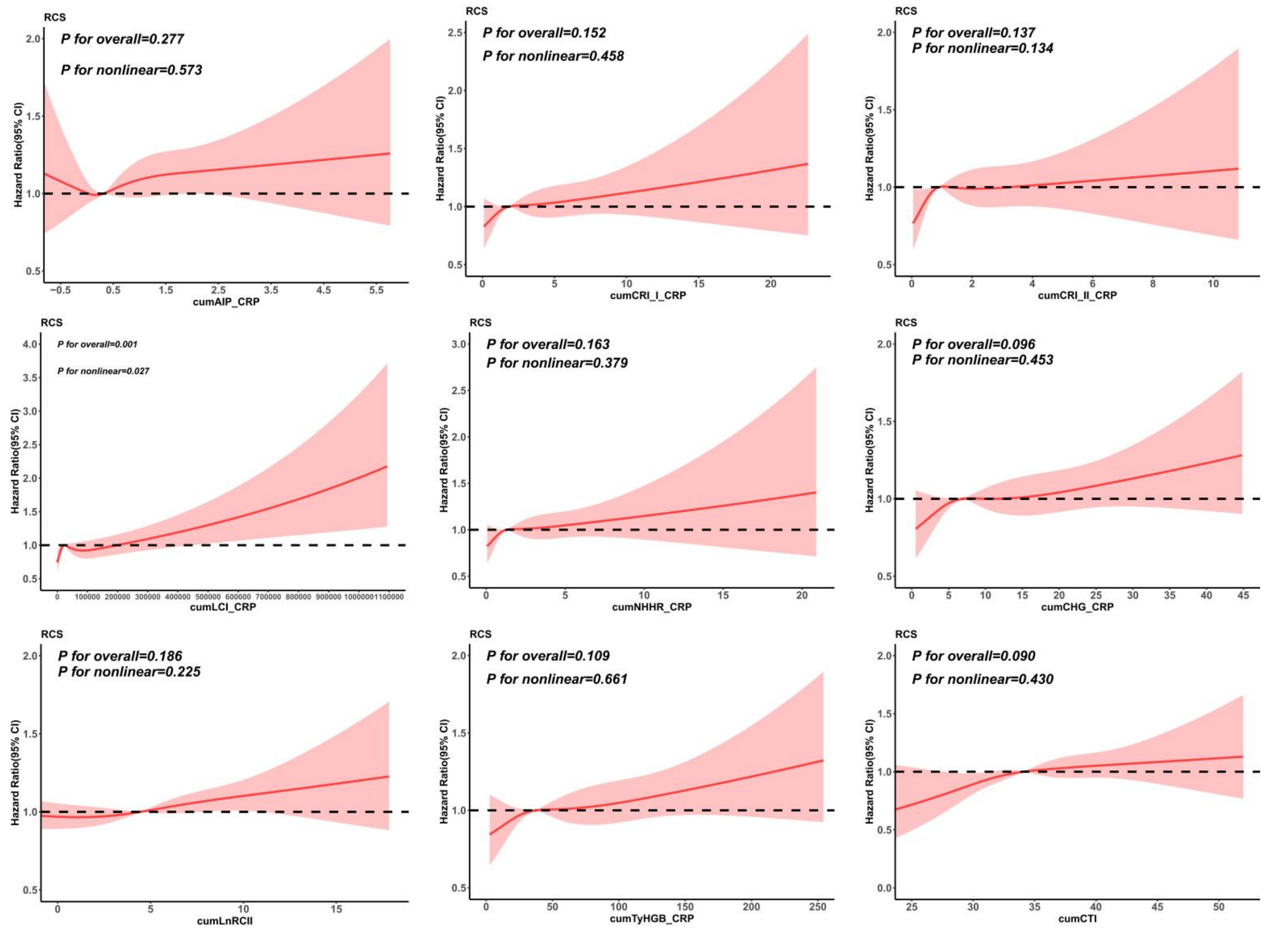

Models were adjusted for age, sex, BMI, SBP, DBP, WC, HR, CR, UA, hypertension, hyperlipidemia, exercise, drink, smoke, and medication use (antihypertensive agents,lipid-lowering drugs)

Fig.S2 Dose-response relationships between nine cumulative lipid-inflammatory indices and IHD risk

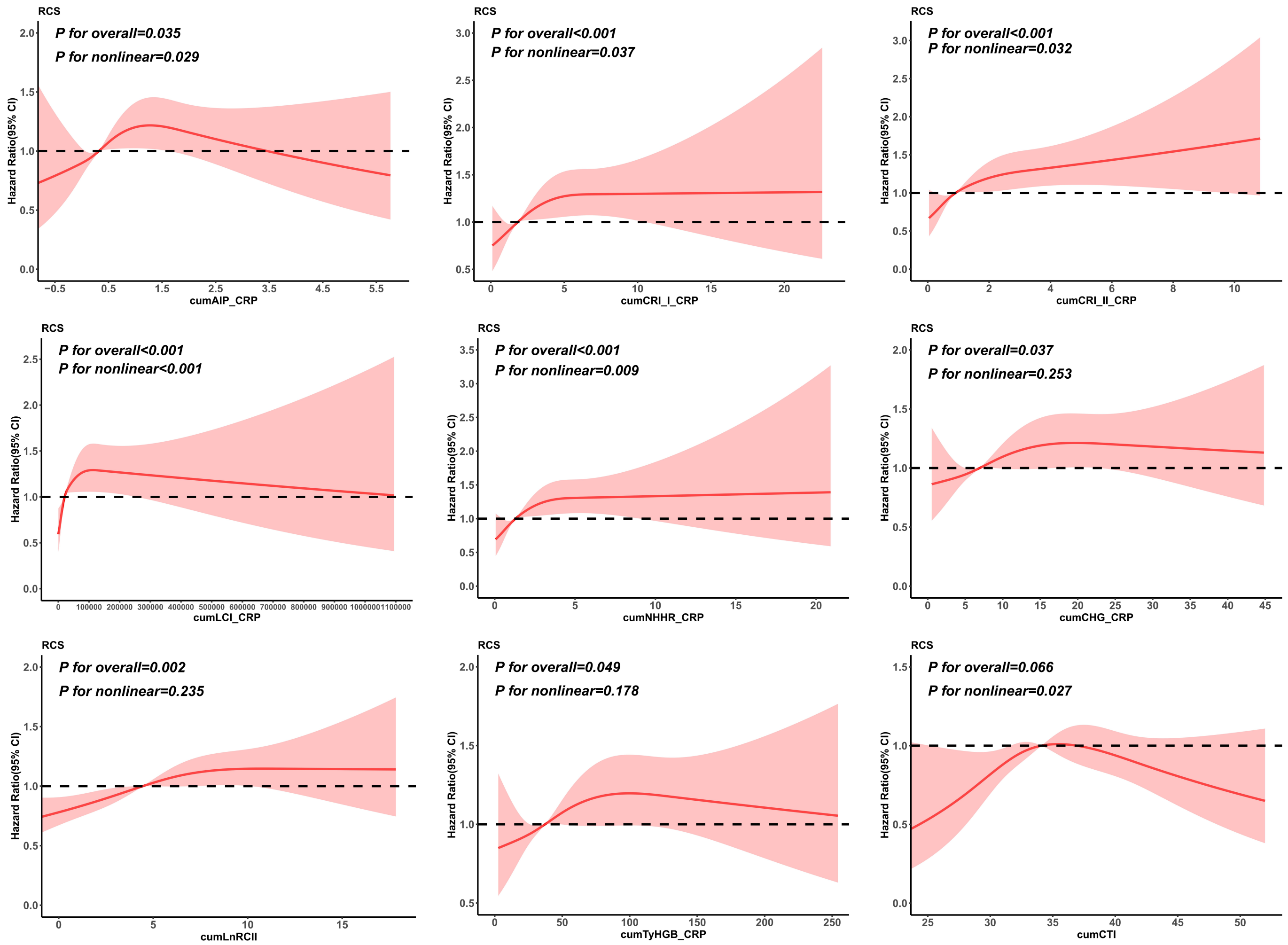

Models were adjusted for age, sex, BMI, SBP, DBP, WC, HR, CR, UA, hypertension, hyperlipidemia, exercise, drink, smoke, and medication use (antihypertensive agents,lipid-lowering drugs)

Fig.S3 Dose-response relationships between nine cumulative lipid-inflammatory indices and T2D risk

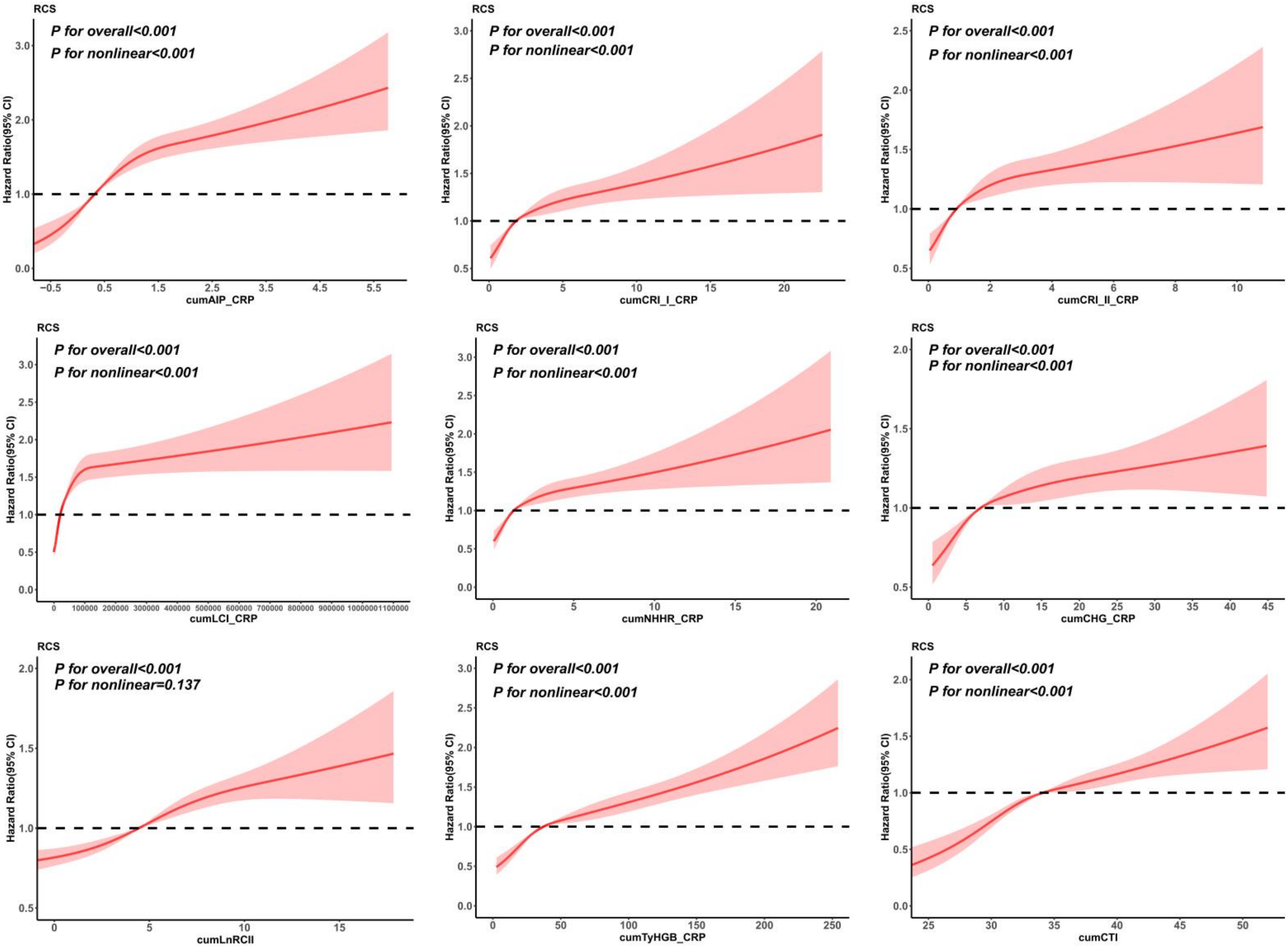

Models were adjusted for age, sex, BMI, SBP, DBP, WC, HR, CR, UA, hypertension, hyperlipidemia, exercise, drink, smoke, and medication use (antihypertensive agents,lipid-lowering drugs)

Fig.S4 Subgroup analyses for the associations of cumulative lipid-inflammatory indices with incident CMM

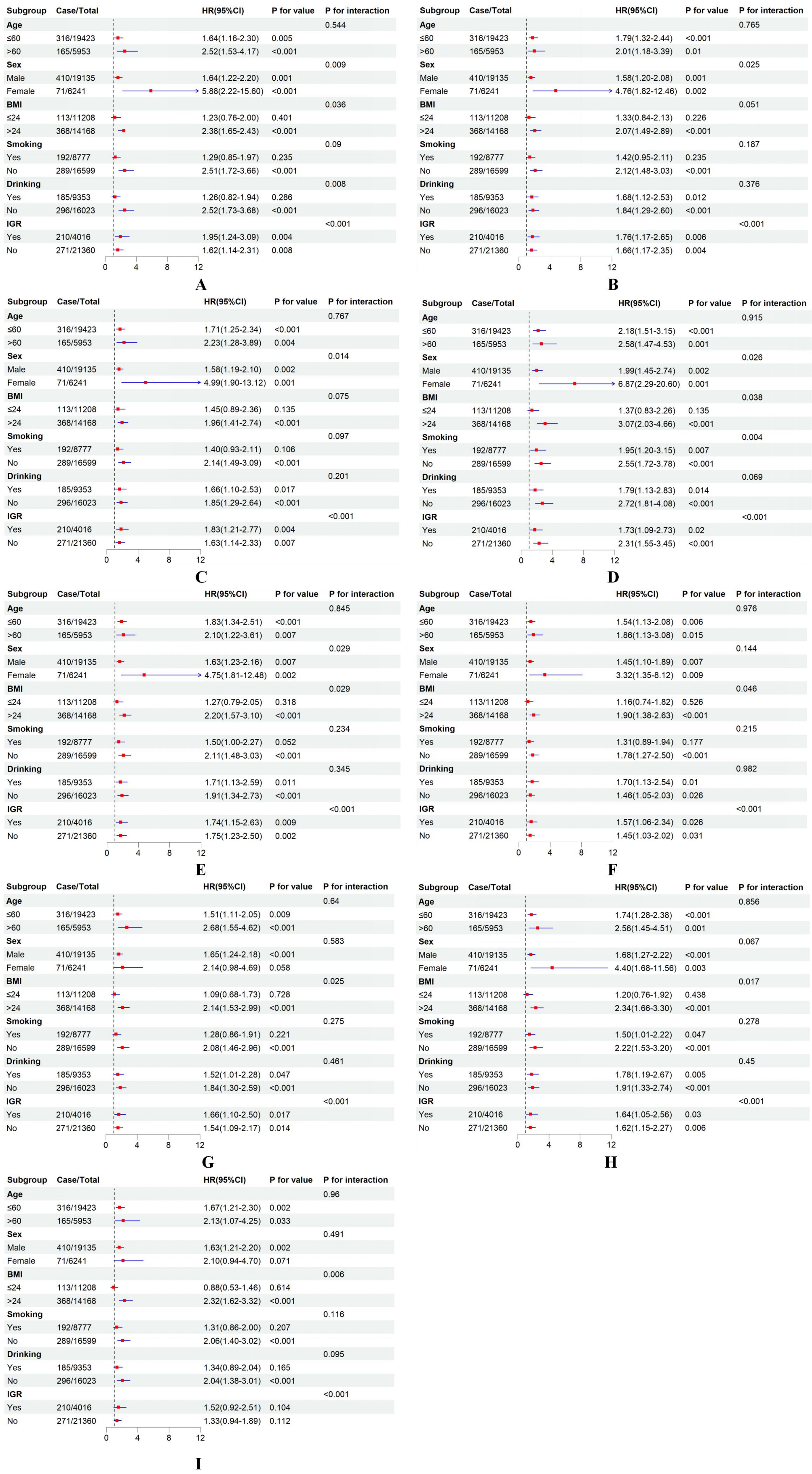

(A) cumAIP_CRP; (B) cumCRI_I_CRP; (C) cumCRI_II_CRP; (D) cumLCI_CRP; (E) cumNHHR_CRP; (F) cumCHG_CRP; (G) cumLnRCII; (H) cumTyHGB_CRP; (I) cumCTI

Models were adjusted for age, sex, BMI, SBP, DBP, WC, HR, CR, UA, hypertension, hyperlipidemia, exercise, drink, smoke, and medication use (antihypertensive agents,lipid-lowering drugs)
